# Oral *Streptococcus salivarius* Couples Neutrophil IRGM1 Signaling to NET Formation and Colorectal Cancer Metastasis

**DOI:** 10.1002/advs.202516546

**Published:** 2026-02-27

**Authors:** Fengyi Liu, Yuan Wang, Hengxuan Cai, Qianwen Yue, Xueli Liu, Xuanwen Bao, Zeng Wang, Xuehan Li, Lai Wei, Zhihua Zhang, Fan Xing, Yi Xu, Xianjun Li, Wei Xu

**Affiliations:** ^1^ Department of Integrated Traditional Chinese and Western Medicine First Affiliated Hospital of Harbin Medical University Harbin P. R. China; ^2^ The Key Laboratory of Myocardial Ischemia Chinese Ministry of Education Harbin P. R. China; ^3^ TCM‐WM Department The Second Affiliated Hospital of Chongqing Medical University Chongqing P. R. China; ^4^ Collaborative Innovation Center for Genetics and Development State Key Laboratory of Genetic Engineering Shanghai Engineering Research Center of Industrial Microorganisms Department of Microbiology School of Life Sciences Fudan University Shanghai P. R. China; ^5^ Department of Medical Oncology The First Affiliated Hospital Zhejiang University School of Medicine Hangzhou P. R. China; ^6^ Key Laboratory of Molecular Oncology in Heilongjiang Province Harbin P. R. China; ^7^ Department of Pathology School of Clinical Medicine Li Ka Shing Faculty of Medicine The University of Hong Kong Hong Kong P. R. China

**Keywords:** colorectal cancer metastasis, IRGM1‐IQGAP1 complex, neutrophil extracellular traps, oral microbiota, *Streptococcus salivarius*, Wnt5a‐PI3K/AKT signaling

## Abstract

Under certain conditions, components of the oral microbiota have been detected in the gastrointestinal tract, implicating oral microbial communities in intestinal immune regulation. However, the mechanisms by which oral microbiota contribute to metastasis at distant organs remain unclear. Here, we demonstrate that the oral bacterium *Streptococcus salivarius* promotes metastasis of colorectal cancer (CRC) by inducing neutrophil extracellular trap (NET) formation. Mechanistically, *S. salivarius* induces formation of an IRGM1‐IQGAP1 complex in neutrophils, in which immune‐related GTPase M1 (IRGM1) interacts with IQ motif‐containing GTPase‐activating protein 1 (IQGAP1), leading to activation of Wnt5a signaling and subsequent engagement of the PI3K/AKT pathway, thereby promoting NET formation. Functional validation experiments show that conditional deletion of IRGM1 or pharmacological inhibition of downstream signaling using XAV‐939 markedly attenuates *S. salivarius‐*induced NET formation, indicating the requirement of this pathway in the metastatic process. Furthermore, clinical sample analyses reveal that *S. salivarius* is significantly enriched in the tongue coating and feces of patients with CRC and is elevated within the tumor microenvironment. Together, these findings identify IRGM1‐IQGAP1‐mediated Wnt5a‐PI3K/AKT signaling as a mechanistic link between oral microbiota and neutrophil‐driven immune responses in cancer metastasis.

## Graphical abstract

The oral bacterium *Streptococcus salivarius* promotes colorectal cancer metastasis by inducing neutrophil extracellular trap (NET) formation. Mechanistically, IRGM1–IQGAP1 interaction activates Wnt5a–PI3K/AKT signaling in neutrophils, driving NET‐mediated tumor progression. These findings reveal a mechanistic link between oral microbiota and immune regulation in cancer metastasis.

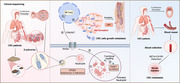

## 1 Introduction

Despite advances in diagnosis and treatment, distant metastasis remains the major cause of mortality in CRC, accounting for over 90% of CRC‐related deaths [1, 2]. While the tumor microenvironment (TME) and gut microbiota are increasingly recognized as important regulators of metastasis [3, 4, 5], whether oral‐origin bacteria contribute to CRC metastasis through immune remodeling remains incompletely understood.

Recent studies have implicated the oral‐gut axis in CRC progression, suggesting that certain pathogenic oral bacteria, such as *Fusobacterium nucleatum (F. nucleatum)*, may colonize intestinal tumors and modulate host immune responses [5, 6]. Notably, neutrophil extracellular traps (NETs)‐chromatin scaffolds decorated with cytotoxic proteins have emerged as double‐edged swords, functioning in pathogen containment while also potentially facilitating tumor cell adhesion and reactivation of dormant metastatic lesions [7, 8].

Several pathogenic bacteria, including *F. nucleatum* and *Streptococcus anginosus (S. anginosus)*, have been reported to promote CRC progression through multiple mechanisms, such as modulation of host immunity, induction of NET formation, and direct interactions with epithelial cells [9, 10, 11, 12]. For instance, *F. nucleatum* has been shown to induce NET formation through innate immune signaling pathways [11, 12], whereas *S. anginosus* may promote bacterial accumulation at premalignant sites via epithelial interactions involving Annexin A [13]. Although NETs are integral to antimicrobial defense, accumulating evidence suggests that they may remodel the extracellular matrix, provide a scaffold for tumor cell migration, and facilitate immune evasion, thereby potentially enhancing metastatic dissemination [7]. However, how oral pathogenic bacteria regulate NET formation in the context of colorectal cancer progression remains poorly understood.

Here, we identify* S. salivarius*, an oral commensal bacterium enriched in the tongue microbiota and tumor tissues of CRC patients, as being associated with pulmonary metastasis. Through a series of experimental analyses, our findings suggest that this process may involve IRGM1‐dependent neutrophil signaling, whereby *S. salivarius* appears capable of translocating across the gut‐lung axis and selectively activating neutrophils. Within neutrophils, IRGM1 may function as a microbial‐sensing mediator by forming a complex with IQGAP1, thereby engaging Wnt5a‐dependent signaling and subsequent PI3K/AKT pathway activation [14, 15, 16], ultimately promoting NET formation. These NETs may, in turn, reshape the local microenvironment and contribute to the establishment of a pro‐metastatic niche that is permissive to CRC cell colonization. Collectively, our findings suggest a potential mechanism by which oral microbiota engage neutrophil‐driven immune pathways to influence CRC metastasis. [17, 18].

## 2 Results

### 2.1 Results 1: Clinical and Microbial Profiling Identify Oral‐Origin *S. salivarius* as a CRC‐Associated Bacterium

**FIGURE 1:**
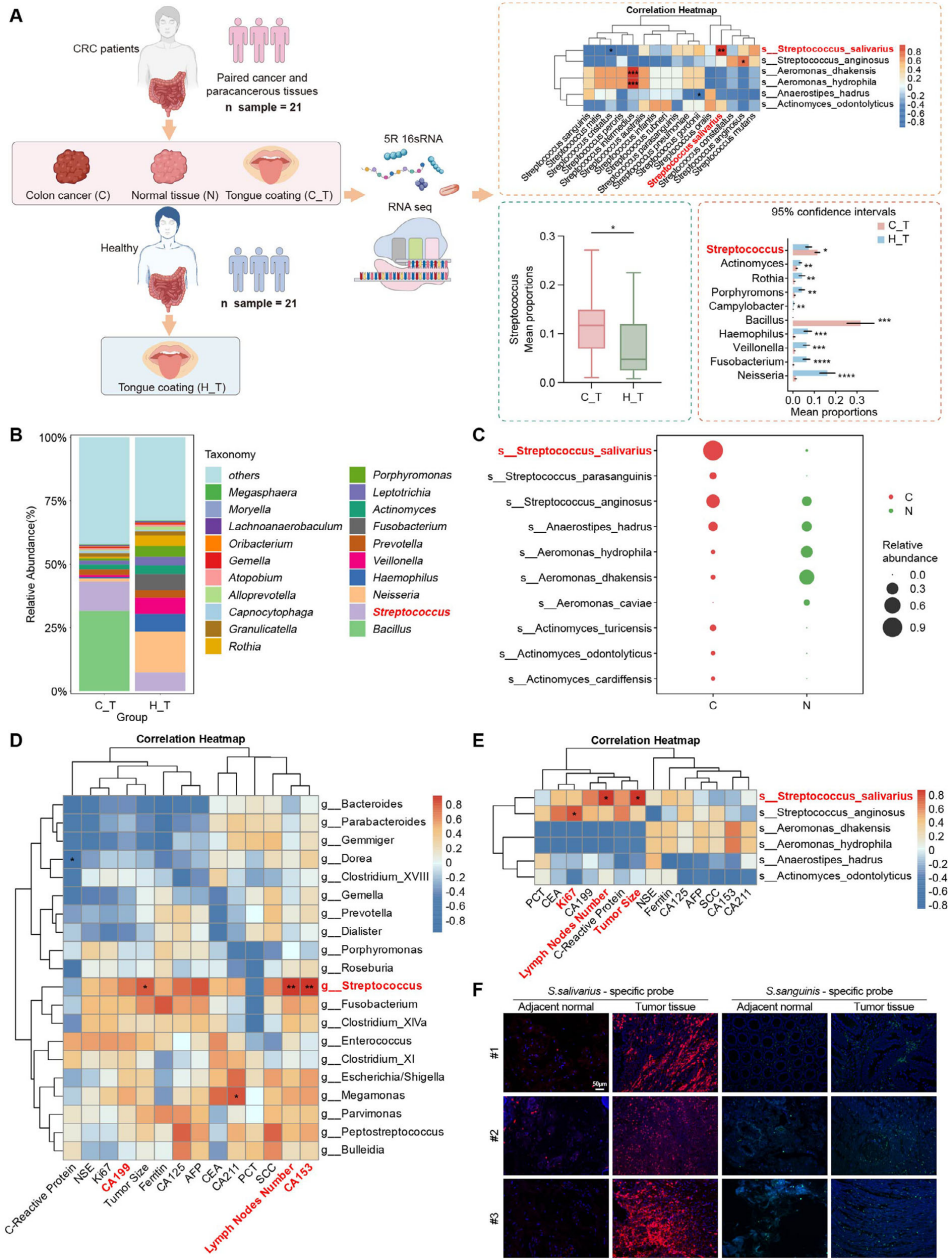
Identification of oral‐gut translocated *S. salivarius* as a metastasis‐associated microbe in CRC. (A) Schematic overview of the study design and analytical workflow, with the right panel summarizing key clinical correlations between tongue‐coating microbiota and tumor‐associated microbiota in patients with CRC. *Schematic illustrations were created using BioRender.com*. (B) Composition of microbial genera in tongue‐coating samples from CRC patients (C_T) and healthy individuals (H_T). The relative abundance of *Streptococcus* is shown. (C) Relative abundance of microbial species in tumor (C) and matched adjacent normal (N) tissues from patients with CRC (n = 21 pairs), showing enrichment of *S. salivarius* (highlighted in red) in tumor samples. Statistical significance was determined using a two‐tailed Wilcoxon matched‐pairs signed‐rank test. (D‐E) Spearman's rank correlation analysis between tumor‐associated microbiota and clinical phenotypes in patients with CRC (D: genus level; E: species level). Correlation coefficients are indicated. *Streptococcus* at the genus level and *S. salivarius* at the species level exhibited positive correlations with metastasis‐related clinical indicators. P values were adjusted for multiple comparisons using the Benjamini–Hochberg false discovery rate (FDR) method (^*^p < 0.05, ^**^ *p* < 0.01). (F) Representative fluorescence in situ hybridization (FISH) images showing the colonization of *S. salivarius* in human CRC tumor and matched adjacent normal tissues. Images are representative of three patients with paired tumor and adjacent normal samples. A Cy3‐labeled probe targeting *S. salivarius* (red) and a FAM‐labeled probe targeting *S. sanguinis* (green) were used. Arrows indicate colonization sites. Scale bar, 50 µm.

To characterize the microbiome across oral and intestinal niches in CRC, we collected tongue‐coating samples from CRC patients and healthy controls, as well as tumor and matched adjacent normal tissues from treatment‐naive CRC patients. Following quality control, 21 high‐quality paired tissue samples, 32 tongue‐coating samples from CRC patients, and 21 tongue‐coating samples from healthy controls were retained for downstream 16S rRNA sequencing and RNA‐seq analyses (Figure 1A; Table S1). Principal component analysis revealed a clear separation between tongue microbiota from CRC patients and healthy controls (Figure S1A), as well as between tumor tissues and matched adjacent normal tissues from CRC patients (Figure S1B). *Streptococcus* was significantly enriched in tongue‐coating samples from CRC patients compared with healthy controls (Figure S1C; Figure 1B). At the species level, *S. salivarius* abundance was markedly elevated in tumor tissues compared with matched adjacent tissues (Figure 1C).

Consistently, 16S rRNA sequencing of fecal samples from 15 CRC patients revealed a significant enrichment of *Streptococcus* (Figure S1D,F), suggesting that its increased abundance is not confined to the TME. To further assess species‐level changes, quantitative PCR analysis of fecal samples from an independent cohort of seven CRC patients and healthy controls showed that *S. salivarius* mRNA levels were markedly elevated in CRC patients (Figure S1E). Alpha diversity analysis revealed significant differences in microbial richness and evenness between tongue‐coating samples from CRC patients and healthy controls, as reflected by the observed species, Simpson, and Shannon indices (Figure S1H).

Clinically, metastatic CRC (mCRC) patients showed larger tumor burden and higher lymph node involvement than non‐metastatic patients (*p < 0.05*; Table S2), both of which correlated with increased *S. salivarius* abundance (Figure 1D,E). To validate these findings, fluorescence in situ hybridization (FISH) was performed on the tumor and matched adjacent normal tissues. *S. salivarius* signals were increased in tumor tissues compared with adjacent normal tissues, whereas *S. sanguinis*, used as a control *Streptococcus species*, showed comparable signals between paired samples (Figure 1F). Gene set enrichment analysis (GSEA) revealing significant enrichment of Gene Ontology biological process terms related to “cellular response to stimulus” and “signal transduction in association” enrichment across CRC samples (Figure S1G). Together, these multi‐site and multi‐platform analyses demonstrate that *S. salivarius* is consistently enriched across oral, fecal, and tumor‐associated niches in CRC patients.

### 2.2 Results 2: Neutrophils are a Key Mediator in *S. salivarius*‐Driven CRC Progression

Before functional analyses, we characterized the in vitro growth properties and strain identity of oral‐derived *S. salivarius*.

**FIGURE 2:**
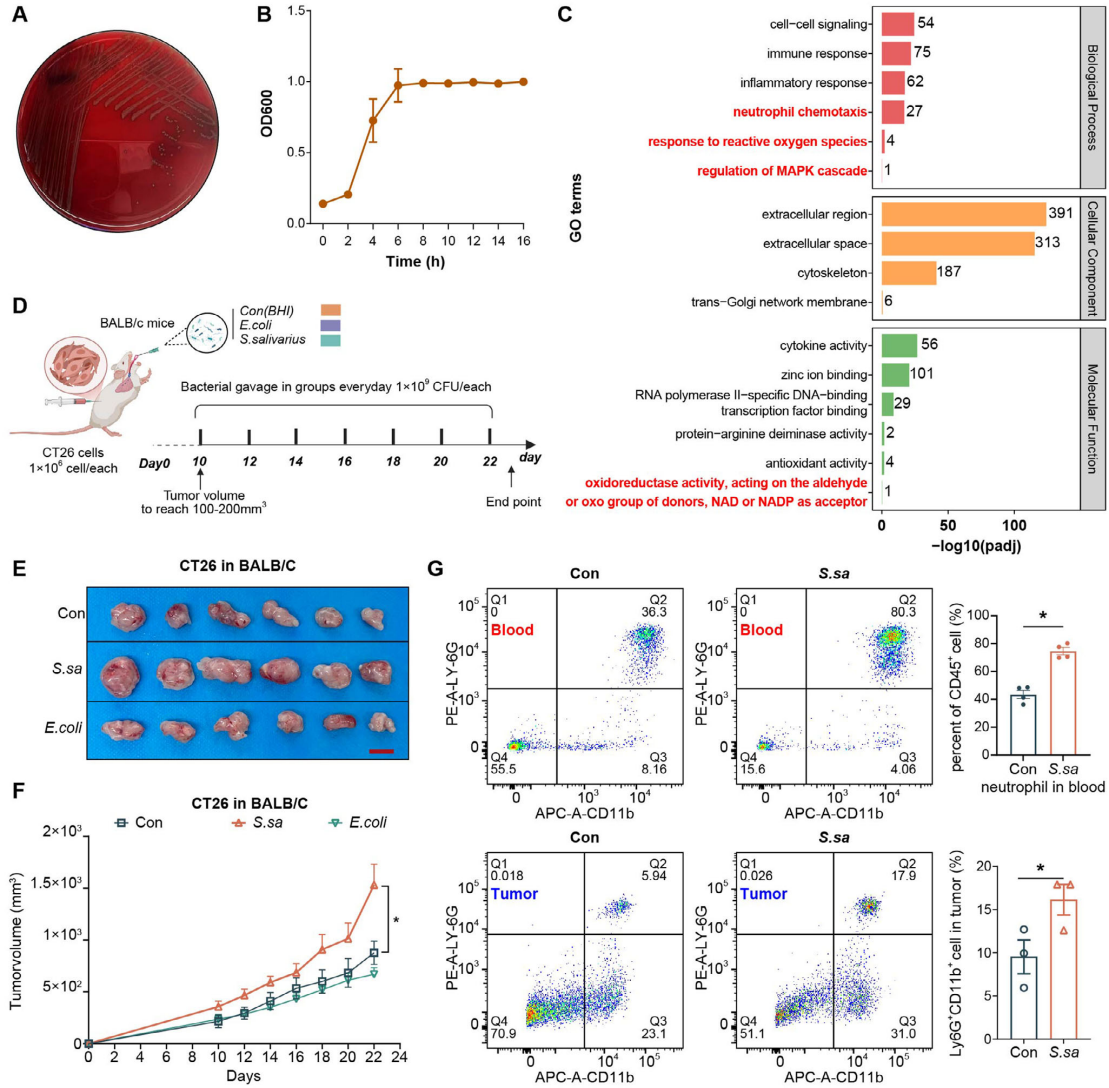
*S. salivarius* increases tumor burden and is associated with neutrophil accumulation in vivo. (A) Representative image of *S. salivarius* colonies grown on blood agar plates. (B) In vitro growth curve of *S. salivarius* measured by optical density at 600 nm (OD600). (C) Gene Ontology (GO) enrichment analysis of upregulated genes identified by bulk RNA sequencing from CRC tumor tissues and matched adjacent normal tissues, highlighting pathways related to inflammation and neutrophil‐associated processes. P values were adjusted for multiple comparisons using the Benjamin‐Hochberg FDR method. (D) Schematic overview of the in vivo experimental design. BALB/c mice were subcutaneously injected with CT26 cells, and oral gavage of *S. salivarius* (1 × 10^9^ CFU), *Escherichia coli*, or BHI was initiated when tumors reached approximately 100 mm^3^ and continued until the experimental endpoint. *Schematic illustration was created using BioRender.com*. (E) Representative images of tumors from control, *S. salivarius*‐treated, and *E. coli*‐treated mice at the experimental endpoint. Scale bar, 1 cm. (F) Tumor volume growth curves showing increased tumor volume in the *S. salivarius*‐treated group compared with controls. Data are presented as mean ± SD (n = 6 mice per group). Statistical significance was determined using two‐way repeated‐measures analysis of variance (ANOVA) with time and treatment as factors, followed by Bonferroni's multiple‐comparison test.(^*^p < 0.05) (G) Flow cytometric analysis of Ly6G^+^CD11b^+^ neutrophils in peripheral blood and tumor tissues from control and *S. salivarius*‐treated mice. Data are presented as mean ± SD. Statistical significance was determined using a two‐tailed Mann‐Whitney U test (^*^p < 0.05, ^**^ *p* < 0.01).

Correlation analysis revealed a strong association between *S. salivarius* abundance in tongue‐coating samples and tumor tissues from CRC patients, prompting further functional investigation (Figure S2A). Before in vivo studies, *S. salivarius* isolates were cultured and characterized in vitro. Growth curve analysis showed that *S. salivarius* reached the logarithmic growth phase within 5 h of culture (Figure 2A,B) and genomic consistency across isolates (Figure S2B–D). Optimization experiments further identified a multiplicity of infection (MOI) of 100 as an appropriate concentration for subsequent functional assays (Figure S2E,F).

To assess the impact of *S. salivarius* on tumor progression in vivo, a subcutaneous CT26 tumor model was established in BALB/c mice (Figure 2D). Oral administration of *S. salivarius* was initiated when tumors reached approximately 100 mm^3^, and tumor growth was monitored longitudinally. Compared with control groups, *S. salivarius ‐*treated mice exhibited increased tumor burden in vivo, as reflected by increased tumor volume over time (Figure 2E,F). Transcriptomic profiling of CRC tumor tissues and matched adjacent normal tissues revealed enrichment of the Net formation pathway. In parallel, multiple NET‐associated signaling pathways, including reactive oxygen species (ROS), peptidylarginine deiminase 4 (PAD4), and the MAPK (Raf/MEK/ERK) cascade, were enriched (Figure 2C). These transcriptomic changes coincided with enrichment of pathways associated with NET formation. Consistent with these transcriptional changes, flow cytometric analysis demonstrated increased accumulation of Ly6G^+^CD11b^+^ neutrophils in both peripheral blood and tumor tissues of *S. salivarius*–treated mice (Figure 2G). In contrast, CD4^+^ and CD8^+^ T cells, macrophages, and other mononuclear immune populations remained largely unchanged (Figure S3D–F). Together, these findings demonstrate that *S. salivarius* exposure increases tumor burden in vivo and is accompanied by selective accumulation of neutrophils, without overt alterations in other immune cell populations.

### 2.3 Results 3: Oral *S. salivarius* Promotes CRC Cell Migration and Distant Metastasis

**FIGURE 3:**
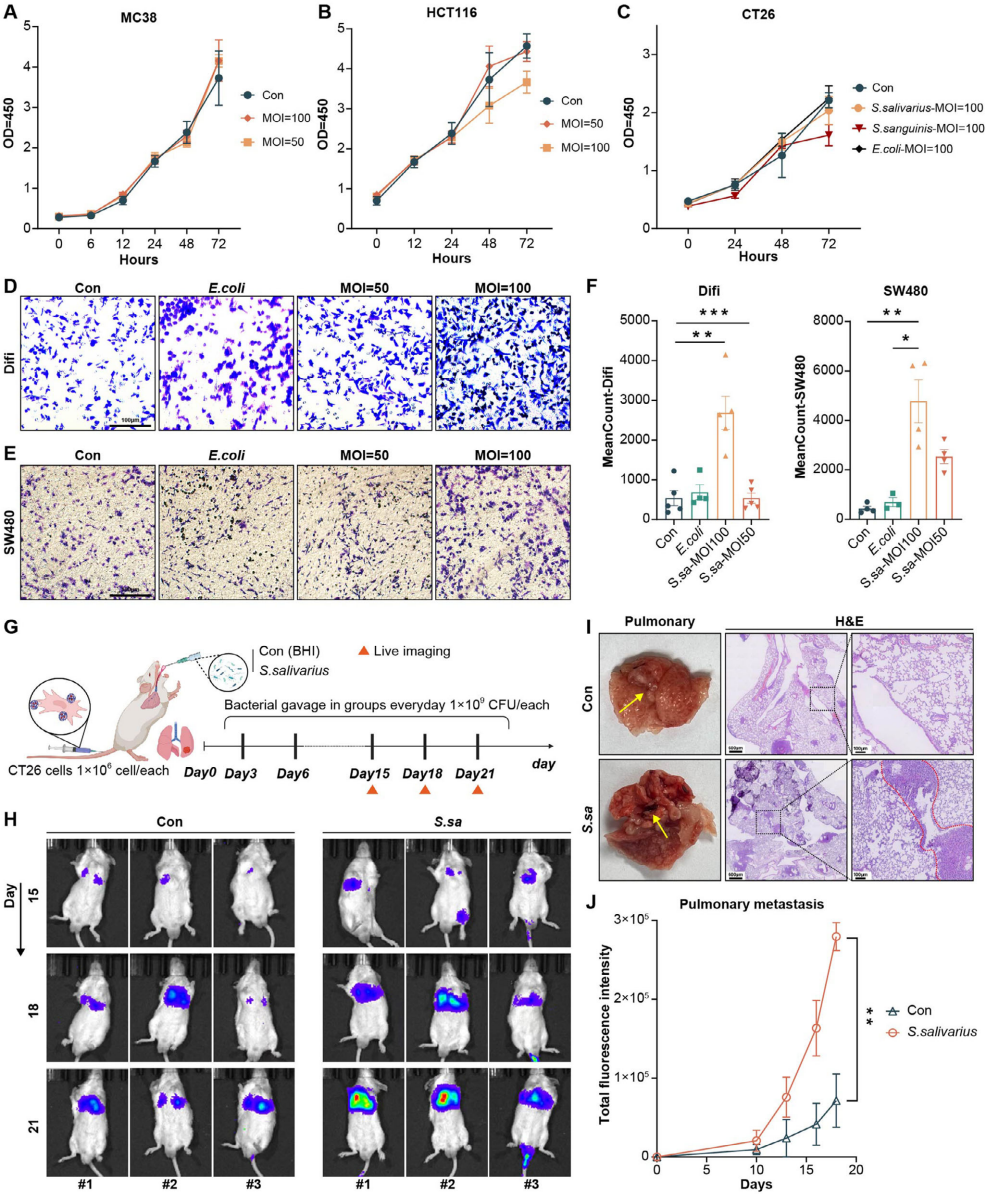
Oral *S. salivarius* facilitates distant colorectal cancer metastasis. (A–C) CCK‐8 proliferation assays showing the effects of *S. salivarius* at different multiplicities of infection (MOI = 50 and 100) on (A) MC38 and (B) HCT116 cells. (C) Comparative CCK‐8 proliferation analysis of CT26 cells co‐cultured with *S. salivarius, S. sanguinis, and E. coli* at MOI = 100. Data are presented as mean ± SD from independent experiments. Statistical significance was determined using two‐way repeated‐measures ANOVA with time and treatment as factors. (D–E) Transwell migration assays showing increased migratory capacity of (D) Difi and (E) SW480 cells following exposure to *S. salivarius* at MOI = 50 and 100. Representative images were acquired using a 20× objective. Scale bar, 100 µm. (F) Quantification of migrated Difi and SW480 cells after co‐culture with *S. salivarius*. Quantification was performed by counting five randomly selected fields per insert and averaging the counts. Data are presented as mean ± SD (n = 4 independent experiments). Planned pairwise comparisons were performed using unpaired two‐tailed Student's *t*‐tests. (G) Schematic overview of the in vivo experimental design.BALB/c mice were injected via the tail vein with CT26‐luc cells and subsequently administered daily oral gavage of *S. salivarius* (1 × 10^9^ CFU) or vehicle control (BHI medium). Bioluminescence imaging was performed on days 15, 18, and 21. *Schematic illustration was created using BioRender.com*. (H) Representative in vivo bioluminescence images showing pulmonary metastatic signals in control and *S. salivarius*‐treated mice. (I) Gross lung morphology and HE staining indicate more invasive tumor architecture following *S. salivarius* treatment. (J) Quantification of pulmonary metastatic burden based on bioluminescence intensity. Data are presented as mean ± SD (n = 3 mice per group). Statistical significance was determined using two‐way repeated‐measures ANOVA with time and treatment as factors. Statistical significance is indicated as^*^ *p* < 0.05, ^**^ *p* < 0.01, ^***^p < 0.001.

Given the observed neutrophil enrichment and tumor microenvironmental alterations associated with *S. salivarius*, we next investigated whether this oral–gut bacterium directly influences the metastatic behavior of CRC cells. Nevertheless, subsequent CCK8 assays showed no notable impact on the proliferation of MC38, HCT116, or CT26 cells across MOIs from 5 to 100 (Figure 3A–C). In contrast, Transwell migration assays revealed a dose‐dependent increase in migratory capacity, with the most pronounced effect observed at MOI = 100 in both Difi and SW480 cells (Figure 3D–F).

To further assess the effect of *S. salivarius* on distant metastatic colonization, an experimental lung metastasis model was established by tail vein injection of CT26‐luc cells into BALB/c mice (Figure 3G). Following tumor cell injection, mice were orally gavaged daily with *S. salivarius* (1 × 10^9^ CFU) or vehicle control (BHI). Pulmonary metastatic progression was monitored longitudinally by in vivo bioluminescence imaging at days 15, 18, and 21 post‐injection. Compared with control mice, *S. salivarius*‐treated mice exhibited progressively increased pulmonary bioluminescent signals over time (Figure 3H). Quantification of total fluorescence intensity confirmed a higher metastatic burden in the lungs of *S. salivarius*‐treated mice (Figure 3J). Consistent with these imaging results, gross examination of excised lungs revealed an increased number of metastatic nodules in *S. salivarius*‐treated mice, which was further validated by hematoxylin and eosin (H&E) staining showing extensive metastatic lesions (Figure 3I).

Collectively, these findings demonstrate that *S. salivarius* selectively enhances CRC cell migration and facilitates distant metastatic colonization in vivo.

### 2.4 Results 4: NET Formation is Associated with *S. salivarius*‐Induced CRC Metastasis

**FIGURE 4:**
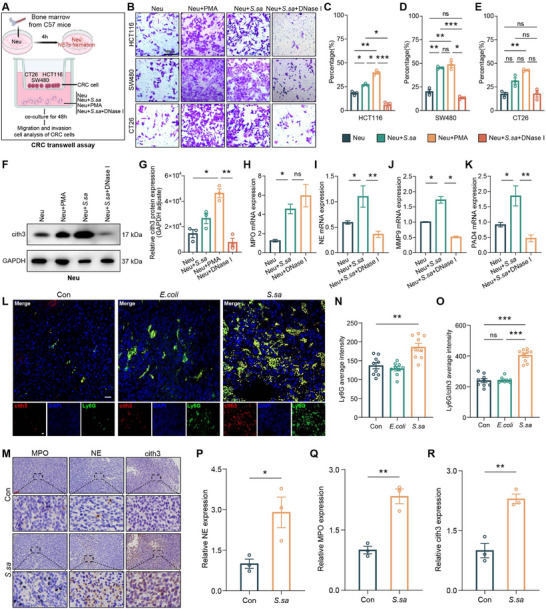
Net formations are associated with *S. salivarius*‐driven CRC metastatic phenotypes. (A) Schematic illustration of the neutrophil‐CRC cell co‐culture transwell migration assay. Mouse bone marrow‐derived neutrophils were seeded in the lower chamber and treated with *S. salivarius*, *S. salivarius plus DNase I*, or *PMA*, while CRC cell lines (CT26, HCT116, and SW480) were placed in the upper chamber. Migration was assessed after 16 h. Schematic illustration was created using BioRender.com. (B) Representative transwell migration images under each condition. Representative images were acquired using a 20× objective. Scale bar, 100 µm. (C–E) Quantification of migrated CRC cells under different treatment conditions. Data are presented as mean ± SD from three independent experiments. Statistical significance was determined using Welch ANOVA followed by Dunnett T3 multiple‐comparison test. (F–G) Immunoblot analysis of citrullinated histone H3 (citH3) in neutrophil‐conditioned media following different stimulation conditions. Protein levels were quantified by densitometry, normalized to GAPDH, and expressed as fold change relative to control. Statistical significance was determined using an unpaired two‐tailed Student’ s *t*‐test. (H–K) Quantitative real‐time PCR (qRT–PCR) analysis of NET‐related genes (*MPO, NE, PAD4, and MMP9*) in neutrophils subjected to the indicated treatments. Statistical significance was determined using Welch ANOVA tests. (L) Representative immunofluorescence images of subcutaneous CRC tumors from mice gavaged with *S. salivarius* or vehicle control, showing co‐localization of citH3 (red) and Ly6G (green). (N–O) Quantification of citH3^+^/Ly6G^+^ double‐positive cells from (L) across ≥3 randomly selected fields per mouse (n = 5 mice per group). Statistical significance was determined using Welch ANOVA. Magnification, 200×. (M–R) Immunohistochemistry staining of tumor sections showing increased enrichment of NETs markers (*MPO, NE, and citH*3) in the tumor microenvironment following *S. salivarius* treatment. Statistical significance was determined using an unpaired two‐tailed Student’ s *t*‐test. Data are presented as mean ± standard deviation (SD) from three independent experiments. ns, not significant; ^*^ *p* < 0.05, ^**^p < 0.01, ^***^ *p* < 0.001.

To evaluate whether *S. salivarius*–induced neutrophil activation promotes CRC cell migration, a Transwell co‐culture system was established (Figure 4A), in which mouse bone marrow‐derived neutrophils were seeded in the lower chamber and exposed to *S. salivarius*, *S. salivarius* plus PMA, or *S. salivarius* plus DNase I, while CRC cells (CT26, HCT116, and SW480) were placed in the upper chamber. CRC cell migration was quantified after 16 h of co‐culture. Compared with neutrophils alone, both PMA‐stimulated neutrophils and neutrophils exposed to *S. salivarius* enhanced CRC cell migration, whereas treatment with DNase I, which degrades extracellular DNA structures, partially attenuated this effect (Figure 4B–E).

To assess whether *S. salivarius* directly triggers NETs release, western blot analysis of citrullinated histone H3 (citH3) was performed in CRC cells exposed to neutrophil‐conditioned medium. Neu + *S. salivarius* markedly increased citH3 expression, while DNase I treatment significantly attenuated this induction (Figure 4F,G). Consistently, qRT–PCR analysis revealed increased expression of NET‐associated genes, including *MPO, NE, PAD4*, and *MMP9*, following *S. salivarius* stimulation, which was attenuated by DNase I treatment (Figure 4H,K).

Immunofluorescence (IF) staining in both in vitro and vivo settings demonstrated abundant NETs deposition in the *S. salivarius* group. In vitro, stimulation of neutrophils with *S. salivarius* induced the formation of characteristic extracellular NET‐like structures, marked by colocalized MPO^+^ and citrullinated histone H3 (citH3) filamentous signals, which were further enhanced by PMA co‐treatment (Figure S5A). Quantitative analysis of fluorescence intensity confirmed a significant increase in NET‐associated markers following *S. salivarius* stimulation, including elevated citH3 and MPO signal intensities compared with control conditions (Figure S5C–F). In vivo, tumor tissues from *S. salivarius*–treated mice exhibited dense citH3^+^/Ly6G^+^ extracellular structures closely associated with tumor cells, whereas DNase I treatment disrupted NET architecture and reduced tumor cell association with neutrophil‐derived structures (Figure 4L–O). Immunohistochemistry (IHC) analysis of tumor sections from CT26 mice further showed significantly elevated *MPO, NE*, and *citH3* levels in the *S. salivarius*‐treated group compared with controls (Figure 4M,P–R).

Collectively, these data indicate that *S. salivarius* robustly activates neutrophils to form NETs, which contribute to increased CRC cell migratory and metastatic phenotypes.

### 2.5 Results 5: *Irgm1* in Neutrophils is a Key Molecular Mediator of *S. salivarius*–Induced CRC Metastasis

**FIGURE 5:**
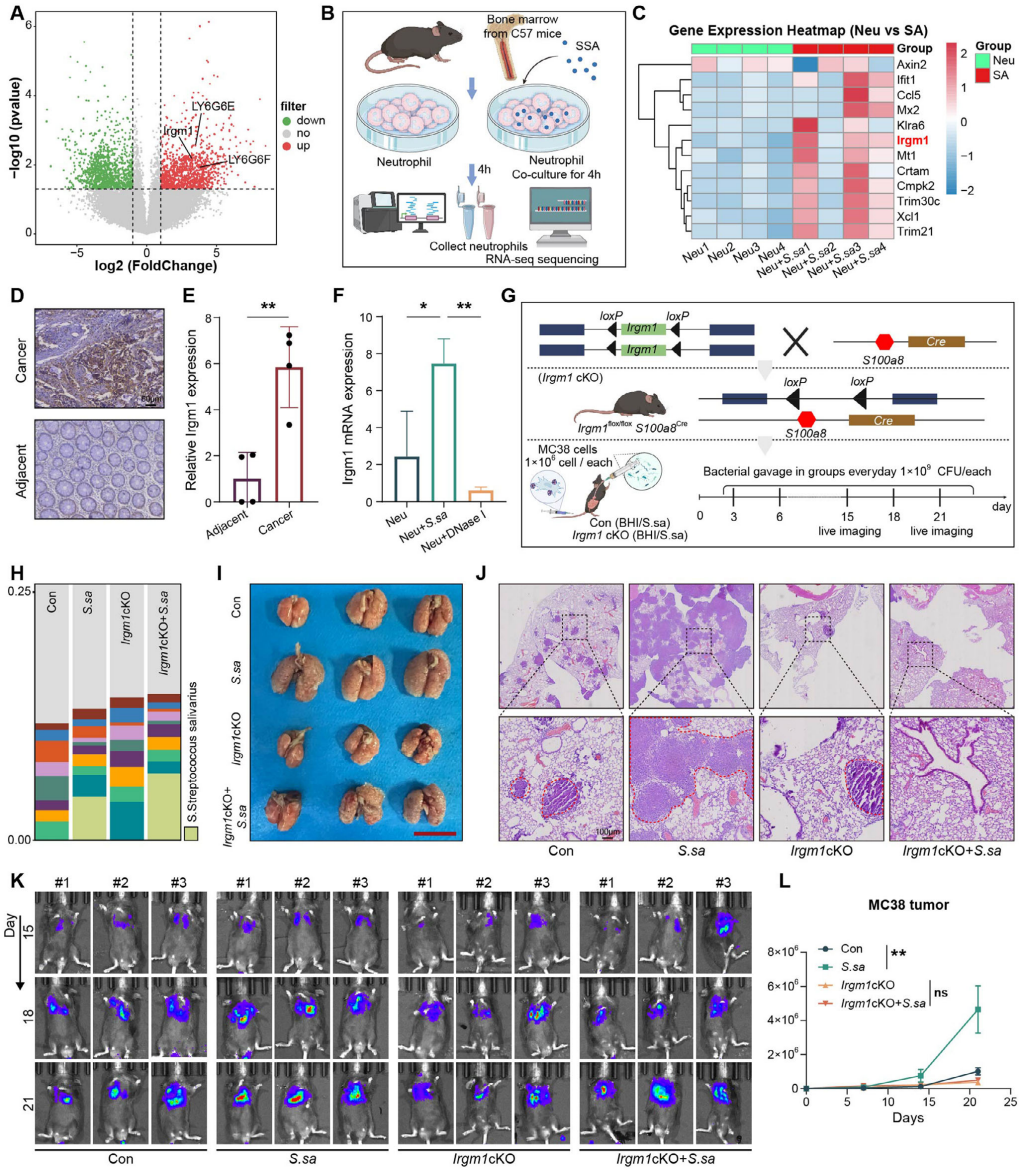
The role of neutrophil‐associated *Irgm1* in tumor metastasis. (A) Volcano plot showing differentially expressed genes between CRC tissues and matched adjacent normal tissues, highlighting the enrichment of immune‐ and neutrophil‐associated genes, including *Irgm1*. (B) Schematic illustration of the experimental workflow. Neutrophils were isolated from mouse bone marrow and co‐cultured with *S. salivarius f*or 4 h before RNA‐seq analysis. Schematic illustration was created using BioRender.com. (C) Heatmap depicting transcriptomic alterations in neutrophils following *S. salivarius* stimulation, with prominent upregulation of *Irgm1*. (D,E) IHC staining further confirmed increased *Irgm1* expression in CRC tumor samples. Statistical significance was determined using an unpaired two‐tailed Student’ s *t*‐test. (F) qRT–PCR analysis of *Irgm1* expression in neutrophils following different treatment conditions. Statistical significance was determined using an unpaired two‐tailed Student’ s *t*‐test. (G) Schematic diagram of neutrophil‐specific *Irgm1‐cKO* mouse model and experimental workflow, including bacterial gavage and in vivo imaging on days 15, 18, and 21. Schematic illustration was created using BioRender.com. (H) Metagenomic analysis of fecal samples confirmed successful colonization of *S. salivarius* in both wild‐type and *Irgm1 cKO* mice. (I–L) Lung metastasis model showing that oral administration of *S. salivarius* significantly increased pulmonary metastatic burden in wild‐type mice, whereas this effect was markedly reduced in neutrophil‐specific *Irgm1* cKO mice. Representative images of (I) gross lung morphology, (J) in vivo bioluminescence imaging, and (K) H&E staining are shown. Quantification of pulmonary metastatic burden (L) is presented as mean ± SD from three independent experiments. Statistical significance was determined using two‐way repeated‐measures ANOVA with time and treatment as factors. ^*^ *p* < 0.05.

To define immune‐related transcriptional changes associated with *S. salivarius*‐driven metastasis, we performed transcriptomic profiling of paired CRC tumors and adjacent normal tissues. Differential expression analysis revealed Irgm1 as one of the most significantly upregulated genes in tumors, together with neutrophil‐associated genes including Ly6g6E and Ly6g6F (Figure 5A). It implicated neutrophil involvement in metastatic progression. In murine bone marrow‐derived neutrophils, stimulation with *S. salivarius* (MOI = 100) robustly induced Irgm1 expression. (Figure 5B,C). Consistently, IHC analysis of CRC patient tissues corroborated the elevated Irgm1 expression levels observed in tumor regions compared with adjacent normal tissues (Figure 5D,E). Moreover, qRT‐PCR analysis of murine neutrophils confirmed that *S. salivarius* stimulation significantly augmented Irgm1 expression, in line with the transcriptomic findings (Figure 5F). In addition, the generation of neutrophil‐specific *Irgm1* conditional knockout mice was confirmed by PCR genotyping and immunoblot analysis (Figure S4C,D).

To assess the functional requirement of Irgm1 in neutrophils, we generated neutrophil‐specific *Irgm1* knockout mice (*Irgm1‐cKO*). Following oral gavage of *S. salivarius*, comparable levels of bacterial colonization were observed in the gastrointestinal tract of *WT* and *Irgm1‐cKO* mice (Figure 5G,H). In a lung metastasis model, *WT* mice treated with *S. salivarius* developed a pronounced metastatic burden, whereas *Irgm1‐cko* mice exhibited a marked reduction in lung metastasis, as shown by lung gross morphology, histological analysis, and bioluminescence imaging over time (Figure 5I–L). Collectively, these results indicate that *Irgm1* expression in neutrophils is required for *S. salivarius*–induced metastatic progression in CRC.

### 2.6 Results 6: *S. salivarius* Induces NET Formation through an IRGM1‐IQGAP1‐Wnt5a Signaling Axis in Neutrophils

Consistent with the transcriptomic differences observed between CRC tissues and matched adjacent normal tissues in Figure 5, KEGG pathway analysis revealed enrichment of Wnt‐related signaling pathways, accompanied by increased expression of pathway‐associated genes such as AXIN2 and WNT2 (Figure S4A,B). To determine whether this Wnt‐related response resulted from a direct effect of *S. salivarius* on tumor epithelial cells, we performed in vitro assays using the human CRC cell line HCT116. Under these conditions, *S. salivarius* treatment did not affect HCT116 cell proliferation and did not measurably alter canonical Wnt/β‐catenin pathway readouts, including *β‐catenin, c‐Myc, and Cyclin D1* protein levels (Figure S4E–H). This suggests that *S. salivarius* does not activate Wnt signaling by directly acting on tumor epithelial cells.

**FIGURE 6:**
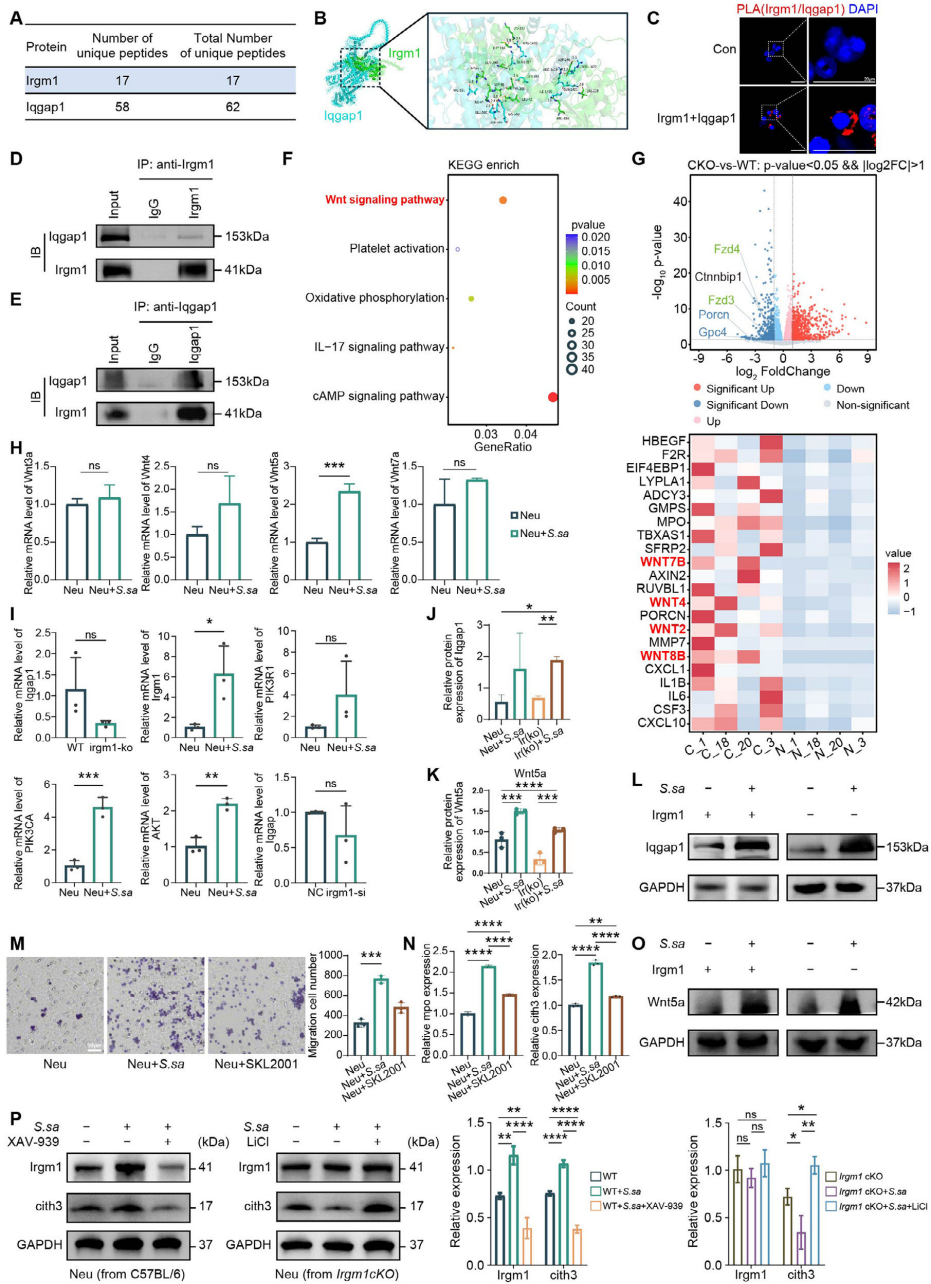
*S. salivarius* induces NET formation through an IRGM1–IQGAP1–Wnt5–PI3K/AKT signaling axis in neutrophils. Immunoprecipitation–mass spectrometry (IP–MS) analysis of control and *Irgm1* conditional knockout neutrophils, identifying differentially associated proteins and unique peptides corresponding to IRGM1 and IQGAP1. (B) Molecular docking analysis demonstrated a high‐confidence interaction between IRGM1 and IQGAP1. (C) Proximity ligation assay (PLA) demonstrates the interaction between Irgm1 and Iqgap1. (D,E) In vitro co‐immunoprecipitation assays confirming the interaction between IRGM1 and IQGAP1. (F) KEGG pathway enrichment analysis of differentially expressed genes from paired colorectal cancer tumor and adjacent normal tissues. Dot size indicates gene count, and color represents adjusted *P* values. (G) Volcano plot and heatmap showing downregulation of Wnt signaling–related genes following *Irgm1* deletion (fold change > 1.5, *P* < 0.05). (H) qRT–PCR analysis of Wnt family gene expression in wild‐type neutrophils following stimulation with *S. salivarius*. (I) qRT–PCR analysis of *Irgm1*, *PIK3CA*, *Akt*, and *Iqgap1* mRNA expression under the indicated conditions. (J–L,O) Western blot analysis showing protein levels of IQGAP1 and WNT5a under the indicated experimental conditions. Protein levels were quantified by densitometric analysis, normalized to the corresponding loading control, and expressed relative to the control. (M) Transwell migration assays demonstrate that *S. salivarius* significantly enhances neutrophil migratory capacity. (N) Enzyme‐linked immunosorbent assay (ELISA) showing increased levels of citH3 and MPO following *S. salivarius* treatment. (P) Immunoblot analysis of IRGM1 and citH3 expression in wild‐type and *Irgm1*‐deficient neutrophils treated with *S. salivarius* in the presence or absence of the Wnt pathway inhibitor XAV‐939 or activator LiCl. For quantitative analyses (H–N,P), data are presented as mean ± SD from three independent experiments. Statistical significance was determined using an unpaired two‐tailed Student's *t*‐test for comparisons between two groups, and ordinary one‐way ANOVA for comparisons among more than two groups. ^*^ *p* < 0.05, ^**^ *p* < 0.01, ^***^ *p* < 0.001.

To elucidate the molecular mechanism by which IRGM1 regulates Net formation, we performed immunoprecipitation‐mass spectrometry (IP‐MS) analysis in IRGM1 conditionally deficient neutrophils and corresponding control cells. Among the differentially associated proteins, unique peptides corresponding to IRGM1 and IQGAP1 were consistently detected (Figure 6A), suggesting a potential interaction between these two proteins.

Molecular docking analysis further predicted a high‐confidence interaction interface between IRGM1 and IQGAP1 (Figure 6B). Proximity ligation assay (PLA) revealed prominent proximity signals between IRGM1 and IQGAP1 within neutrophils (Figure 6C). Consistently, co‐immunoprecipitation assays confirmed a physical association between IRGM1 and IQGAP1 in vitro (Figure 6D,E), indicating that IRGM1 and IQGAP1 form a stable protein complex.

We next investigated the downstream consequences of IRGM1 deficiency on neutrophil signaling programs. KEGG enrichment analysis revealed significant alterations in Wnt‐related signaling pathways when comparing transcriptomes between colorectal cancer tumor tissues and adjacent normal tissues (Figure 6F). Furthermore, volcano plot and heatmap analyses of transcriptome comparisons between IRGM1‐conditionally knocked‐out neutrophils and wild‐type neutrophils revealed synergistic downregulation of multiple Wnt pathway‐associated genes following IRGM1 loss (Figure 6G), suggesting IRGM1 participates in regulating Wnt pathway‐related transcriptional activity in neutrophils.

To identify the specific Wnt ligand involved, wild‐type murine neutrophils were exposed to *S. salivarius* in vitro, followed by quantitative PCR analysis of Wnt family members. Among the genes examined, Wnt5a exhibited a marked and selective upregulation in response to *S. salivarius* stimulation (Figure 6H). Although IRGM1 deletion did not reduce Iqgap1 transcript levels, Wnt5a‐associated signaling was markedly impaired in IRGM1‐deficient neutrophils. Immunoblot analyses further demonstrated that S. salivarius exposure increased the protein levels of IQGAP1 and WNT5a (Figure 6L–O). qPCR analysis of lung tissues from wild‐type and Irgm1‐deficient mice showed that *S. salivarius* exposure was associated with increased expression of *Wnt5a, Iqgap1, Pik3r1, Akt1*, and the NET‐associated marker *citH3*, whereas these responses were attenuated in Irgm1 knockout mice (Figure S6A). In parallel, analysis of subcutaneous tumor tissues revealed elevated mRNA levels of Irgm1, Wnt5a, and PI3K/AKT‐related genes following *S. salivarius* treatment (Figure S6B). Immunoblotting analyses using isolated neutrophils further showed increased protein levels of PI3K and AKT upon *S. salivarius* stimulation, which were diminished with Irgm1 deficiency (Figure S6C–E). Collectively, these data support that *S. salivarius*–associated activation of Wnt5a–PI3K/AKT signaling occurs at both tissue and cellular levels and is dependent, at least in part, on IRGM1.

Functionally, *S. salivarius* stimulation increased neutrophil migratory capacity in a transwell assay and elevated NET‐associated markers CitH3 and MPO (Figure 6M,N). Pharmacological inhibition of Wnt signaling using XAV‐939 reduced *S. salivarius*–associated induction of CitH3 (and IRGM1, where applicable), whereas activation of Wnt signaling with LiCl did not alter Irgm1 expression (Figure 6P). Notably, LiCl partially rescued the impaired NET formation observed in IRGM1‐deficient neutrophils (Figure 6P), consistent with IRGM1 acting upstream of Wnt‐associated signaling in this context. Although changes in nuclear β‐catenin and its target genes were also observed under certain conditions (Figure S6F–I), the genetic and pharmacological data support Wnt5a‐associated signaling as the principal pathway contributing to NET formation. Together, these findings support a model in which *S. salivarius* promotes NET formation through an IRGM1‐IQGAP‐Wnt5a‐PI3K/AKT‐associated signaling axis in neutrophils.

### 2.7 Result 7: *S. salivarius* Reshapes the TME by Activating the Neutrophil IRGM1‐IQGAP1‐WNT5 Axis

**FIGURE 7:**
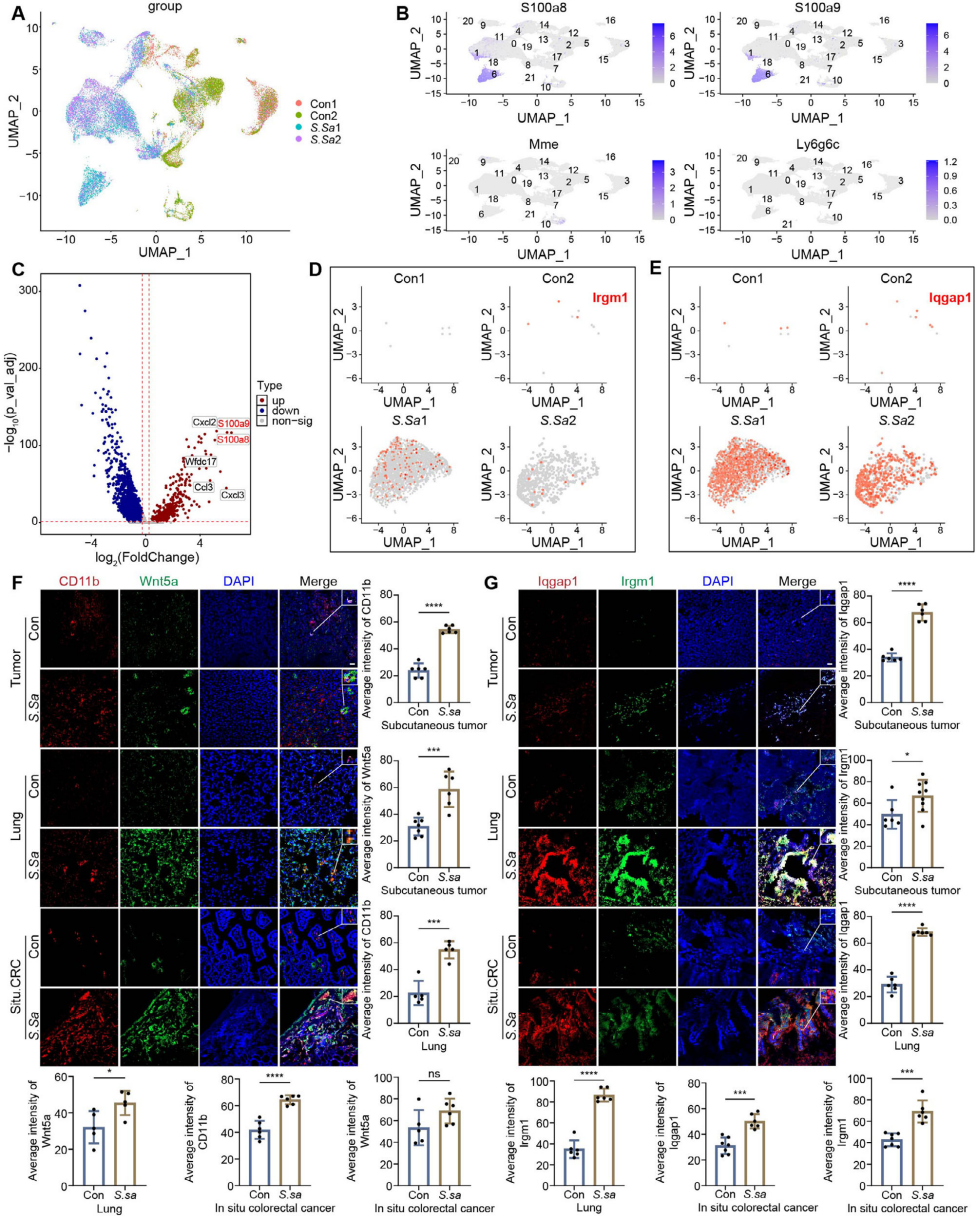
*S. salivarius* reshapes the tumor immune microenvironment and activates the IRGM1–IQGAP1–WNT5 axis. (A) UMAP visualization of single‐cell RNA sequencing data showing the global cellular landscape of tumor samples from control groups (*Con1, Con2*) and S. salivarius–treated groups (*S.sa1*, *S.sa2*). (B) Feature plots displaying the expression patterns of immune cell marker genes *S100a8, S100a9, Mme*, and *Ly6g6c* across UMAP clusters, enabling annotation of neutrophil and related immune cell populations. (C) Volcano plot illustrating differentially expressed genes between control and *S. salivarius*‐treated samples. Significantly upregulated genes include inflammatory and signaling‐related markers such as *Cxcl2, S100a8, Wnt5a, Irgm1*, and *Cxcl3* (adjusted P < 0.05). (D,E) Feature plots showing the distribution of (D) *Iqgap1* and (E) *Irgm1* expression across UMAP clusters in control and S. salivarius–treated groups. (F) Immunofluorescence staining of subcutaneous tumors, lung metastases, and orthotopic colorectal tumors showing CD11b (red), Wnt5a (green), and nuclei (DAPI, blue). Quantification of fluorescence intensity is shown on the right. (G) Immunofluorescence staining of the same tumor models showing Iqgap1(red), Irgm1 (green), and nuclei (DAPI, blue), with corresponding quantitative analysis. Data are presented as mean ± SD. Statistical significance was determined using an unpaired two‐tailed Student's t‐test. ^*^ *p* < 0.05, ^**^ *p* < 0.01, ^***^ *p* < 0.001.

To investigate the role of *S. salivarius* in modulating the CRC tumor microenvironment, we performed an integrated multi‐omics analysis combining single‐cell RNA sequencing (scRNA‐seq) and IF imaging. UMAP visualization revealed marked differences in global clustering patterns between control groups (Con1, Con2) and *S. salivarius*‐treated groups (*S.sa1, S.sa2*), indicating substantial alterations in cellular heterogeneity within the TME (Figure 7A).

Annotation of immune cell populations based on established marker genes, including *S100a8, S100a9*, and *Ly6g6c* (Figure 7B). Differential expression analysis further identified significant upregulation of multiple inflammatory and signaling‐related genes, including *Cxcl2, S100a8, Irgm1*, and *Cxcl3*, in the *S. salivarius*‐treated group compared with controls (Figure 7C). The expression dynamics of Irgm1 and Iqgap1 across experimental groups are shown, supporting the involvement of this signaling axis in *S. salivarius*–associated immune remodeling (Figure 7D,E).

To determine whether these transcriptional changes were reflected at the tissue level, *S. salivarius* was orally administered to mice bearing subcutaneous tumors, lung metastases, or orthotopic colorectal tumors, followed by IF analysis. Across all models, *S. salivarius* treatment resulted in increased infiltration of CD11b^+^ myeloid cells and enhanced fluorescence intensity of *Wnt5a, Iqgap1*, and *Irgm1* within tumor tissues (Figure 7F,G), indicating activation of the IRGM1‐IQGAP1‐WNT5 axis in vivo.

To further substantiate the localization of this signaling axis within neutrophils, IF and proximity ligation assays were performed in mouse bone marrow‐derived neutrophils. Wnt5a and Iqgap1 were readily detected in neutrophils and exhibited co‐localization with Irgm1, while proximity ligation analysis confirmed close spatial association between Irgm1 and Iqgap1 at the cellular level (Figure S5G–L).

Finally, we assessed the association between these molecular changes and tumor phenotypes in vivo and in clinical samples. Western blot analysis of paired colorectal cancer and adjacent normal tissues revealed altered expression of EMT‐related markers and NET‐associated proteins in tumor tissues (Figure S5M). Consistently, *S. salivarius* treatment induced similar changes in EMT markers and citH3 levels in subcutaneous tumor–bearing mice (Figure S5N). Effective colonization of *S. salivarius* was confirmed in the orthotopic colorectal cancer model (Figure S5O), providing a physiological context for its tumor‐modulating effects. Moreover, quantitative PCR analyses of lung and subcutaneous tumor tissues demonstrated significant changes in transcripts associated with Wnt5a, Iqgap1, Pik3r1, and citH3 following *S. salivarius* treatment (Figure S6A,B). Collectively, these data indicate that *S. salivarius* reshapes the colorectal tumor microenvironment by activating the neutrophil IRGM1‐IQGAP1‐WNT5 signaling axis, which is associated with EMT‐related and tumor‐promoting phenotypes.

## 3 Discussion

This study was motivated by a consistent clinical observation that *S. salivarius* is enriched in both the oral cavity and intestinal microbiota of patients with CRC. In addition to its high abundance in tongue coatings, *S. salivarius* was detectable in tumor tissues, with levels that appeared to increase alongside tumor size, lymph node involvement, and metastatic stage. These associations raise the possibility that oral commensal bacteria may actively contribute to CRC progression, rather than merely reflecting disease status.

Our data suggest that *S. salivarius* may be associated with lung metastasis through a neutrophil‐dependent IRGM1‐IQGAP1‐WNT5A‐NETs signaling axis. Exposure to *S. salivarius* was associated with increased IRGM1 expression in neutrophils, enhanced NET formation, and accelerated metastatic dissemination across multiple in vivo models. Integration of single‐cell transcriptomic analyses with in vivo imaging and functional inhibition experiments further supports a link between microbial stimulation, neutrophil activation, and metastatic progression, although the precise causal relationships warrant continued investigation [19].

At the molecular level, IQGAP1 emerged as a central component of this response. IQGAP1 is known to function as a scaffold protein that organizes PI3K signaling in immune cells [20]. In this context, we observed that *S. salivarius* selectively increased expression of the non‐canonical Wnt ligand WNT5A, without activating canonical Wnt signaling in tumor epithelial cells. Previous studies have demonstrated a direct functional association between WNT5A signaling and cytoskeletal regulatory pathways involved in cell migration and polarity, with IQGAP1 identified as a key regulatory factor in this process [21]. These findings suggest that WNT5A may couple microbial‐derived signals to intracellular signaling and cytoskeletal remodeling through IQGAP1.

WNT5A has also been reported to intersect with the PI3K/AKT pathway and to participate in immune cell activation and inflammatory responses [22, 23]. Consistent with these reports, we observed concurrent induction of WNT5A, activation of IQGAP1, and increased PI3K/AKT signaling in neutrophils. Given the established role of PI3K/AKT signaling in neutrophil activation and NET formation [24, 25], these data support a model in which IQGAP1 integrates WNT5A‐associated upstream signals and relays them to PI3K/AKT, thereby facilitating NET release [26, 27, 28].

From a clinical perspective, these findings may have implications beyond mechanistic insight. Metastasis remains the principal driver of mortality in colorectal cancer, yet current risk stratification approaches rarely incorporate microbiota‐associated immune states. In this context, the enrichment of *S. salivarius* in tongue coatings and tumor tissues, together with its association with neutrophil activation and metastatic burden, suggests that this organism may reflect a biologically meaningful disease‐associated microbial signature rather than an incidental bystander. Notably, our previous work based on tongue‐coating analyses revealed that colorectal cancer progression is accompanied by characteristic metabolic alterations, particularly lipid metabolic dysregulation, reflecting tumor microenvironment‐associated metabolic remodeling [29, 30]. These findings further support the notion that oral microbial and metabolic features captured at the tongue surface may systematically mirror disease‐relevant tumo‐microbiome interactions rather than isolated local changes. Detection of *S. salivarius* in oral or tumor‐associated samples may therefore represent a low‐burden adjunct to existing clinical assessments, particularly for identifying patients at risk of aggressive disease [31, 32].

Equally important is the host immune response highlighted in this study. The IRGM1‐centered neutrophil program provides a plausible link between microbial exposure and metastatic competence, framing metastasis, at least in part, as an immune‐conditioned process. Rather than targeting bacteria directly, an approach that may be limited by microbial variability and transience‐modulation of the IRGM ‐Wnt‐NETs signaling axis could offer a more stable therapeutic entry point. Such strategies may be particularly relevant in tumors characterized by prominent neutrophil infiltration and inflammatory remodeling [32, 33, 34, 35].

Nevertheless, the broader clinical relevance of IRGM1 requires further validation. Although our data support its involvement in metastatic progression, establishing its prognostic or predictive value will depend on analyses in larger, independent patient cohorts with longitudinal follow‐up. Future studies integrating multi‐center clinical datasets, immune profiling, and microbial assessments will be essential to determine whether IRGM1‐associated signatures can meaningfully inform patient stratification or therapeutic decision‐making [36, 37]. Taken together, these observations highlight a potentially actionable intersection between the oral–gut microbiome, innate immune programming, and colorectal cancer metastasis, and point toward new directions for microbiome‐informed precision oncology.

## 4 Experimental Model and Study Participant Details

### 4.1 Materials

Materials/reagents are listed with catalogue numbers in Tables S1 and S2.

### 4.2 Patients and Clinical Samples

Tumor tissues, adjacent non‐tumor tissues, and tongue coating samples from 21 patients with CRC were collected for this study between September 2022 and May 2023 at the First Affiliated Hospital of Harbin Medical University. Written informed consent was obtained from all participants, and the protocol was approved by the Ethical Committee of the First Hospital, Harbin Medical University (No. 2022151). The summary of patient characteristics is in Table S1.

### 4.3 Cell Culture and Bacterial Strain Culture

Human CRC cell lines HCT116 (RRID: CVCL_0291) and SW480 (RRID: CVCL_0546), along with murine CRC cell line CT26 (RRID: CVCL_7254), were used in this study.

CT26 and Luc‐CT26 cells were obtained from Qida Bio (Guangzhou, China; Catalog No. CD0593), and Luc‐MC38 cells were purchased from the same supplier (Catalog No. CD0641). HCT116 was purchased from Procell Life Science (Wuhan, China; Catalog No. CL‐0096), and SW480 and wild‐type CT26 were sourced from the American Type Culture Collection (ATCC).

CT26 and Luc‐CT26 cells were maintained in RPMI‐1640 medium supplemented with 10% fetal bovine serum (FBS), while HCT116, SW480, and Luc‐MC38 cells were cultured in DMEM containing 10% FBS. All cell lines were cultured at 37°C in a humidified atmosphere with 5% CO_2_.

All cell lines were authenticated by short tandem repeat (STR) profiling and were confirmed to be free of Mycoplasma contamination using the MycoAlert Mycoplasma Detection Kit (Lonza Bioscience, Basel, Switzerland).

*S. salivarius* (BNCC134928), *Escherichia coli* DH5α (*E. coli*, BNCC353719), and *Streptococcus sanguinis* (BNCC354356) strains were purchased from BNCC. The culture conditions for Sa, *E. coli*, and *S. sanguinis* were adapted from previously described protocols. Bacteria were cultured in a brain heart infusion (BHI) medium at 37°C under microaerophilic conditions. After culturing, bacteria were harvested by centrifugation at 5000 rpm for 10 min, washed twice with sterile PBS, and their concentration was adjusted to an OD600 of 1 (approximately 10^9^ CFU/mL).

### 4.4 Mice

Male C57BL/6 and BALB/c mice were procured from Charles River Laboratory Animal Technology Co., Ltd. *Irgm1^flox/flox^* and *S100a8‐cre* mice were obtained from Cyagen Biosciences. To generate neutrophil‐specific *Irgm1*‐deficient mice (*Irgm1^flox/flox^S100a8‐cre*, referred to as *Irgm1‐cKO*), *Irgm1^flox/flox^* mice were crossed with *S100a8‐cre* mice. For the CT26 subcutaneous tumor model, BALB/c mice were used, as CT26 is a syngeneic murine colorectal cancer cell line derived from this strain, thereby ensuring efficient tumor engraftment and intact immune compatibility.

For the experimental lung metastasis model, MC38 cells were injected via the tail vein into C57BL/6 mice, reflecting the syngeneic origin of the MC38 cell line and enabling appropriate host–tumor immune interactions. In experiments involving *Irgm1*‐deficient mice, we used the C57BL/6 background, as the *Irgm1‐cko* line was established and maintained on this genetic background. All mice were maintained under pathogen‐free conditions at the Second Affiliated Hospital of Harbin Medical University. They were housed in a controlled environment with a 12‐h light/dark cycle, constant temperature and humidity, and had ad libitum access to food and water, with no more than five mice per cage. To reduce baseline variation in gut microbiota composition, mice from different experimental groups were co‐housed for two weeks before treatment. Co‐housing facilitates microbial exchange through natural behaviors and is commonly used to equilibrate gut microbiota among experimental groups. All experimental procedures were approved by the Animal Care and Use Committee of the Second Affiliated Hospital of Harbin Medical University (NO.2021‐034).

### 4.5 Animal Experiments

For the subcutaneous tumor model, 1 × 10^6^ CT26 cells were injected into the flanks of BALB/c mice. Once tumor volumes reached 100–150 mm^3^, mice were randomly assigned to three groups and received daily oral gavage of 200 µL bacterial suspension containing 1 × 10^9^ CFU of either *S. salivarius* or *E. coli* for 14 days.

For the experimental lung metastasis model, C57BL/6 mice were injected via the tail vein with 1 × 10^6^ MC38‐luc cells. To evaluate the effects of bacterial intervention, mice were orally administered 200 µL of S. salivarius suspension (10^9^ CFU) daily for 21 consecutive days. At the study endpoint, lung tissues were harvested for histological examination and imaging analyses. Bio luminescence imaging in vivo was conducted every three days using the IVIS bioimaging system, employing VivoGlo Luciferin (Promega) as the imaging substrate. Mice were regularly monitored for changes in tumor size. After 21 days, the mice were euthanized, and their tumors and lungs were harvested for histopathological examination.

### 4.6 Neutrophil Isolation and Stimulation

Bone marrow–derived neutrophils were isolated as described above. The cells were seeded in 6‐well plates at a density of 1 × 10⁶ cells per well and cultured in DMEM at 37°C. Neutrophils were stimulated with phorbol 12‐myristate 13‐acetate (PMA, 1 µM; Beyotime, S1819) to induce NET formation or treated with DNase I (100 U/mL; Beyotime, D7073) for 4 h. In some experiments, neutrophils were exposed to Streptococcus salivarius at a multiplicity of infection (MOI) of 100:1. After incubation, cells or culture supernatants were collected for subsequent quantitative PCR, Western blotting, or immunofluorescence analysis.

### 4.7 Conditioned Medium

Neutrophils were isolated and placed into 6‐well plates, then exposed to *S. salivarius* at an infection ratio of 100:1, PMA (1 µm; S1819; Beyotime), or DNase I (100 U/mL; Beyotime, #D7073) in DMEM for 4 h at 37°C. After incubation, the supernatant was harvested. It was then centrifuged to eliminate bacteria, cells, and debris, after which different conditioned media (CM) were collected and preserved at −80°C for subsequent experiments.

### 4.8 IHC and H&E Staining

IHC was utilized on paraffin‐embedded tumor samples to identify the presence of MPO, NE, citH3, and Irgm1. After deparaffinization, the slides were autoclaved in sodium citrate for antigen retrieval, followed by incubation with 1% hydrogen peroxide to quench endogenous peroxidase activity, and blocked with 3% BSA. The slides were then incubated overnight at 4°C with primary antibodies. Signals were developed using Liquid DAB+ Substrate Chromogen (Dako, USA). To determine the proportion of positive cells, at least 1000 cells were counted across five randomly selected microscopic fields for each sample. All cases were evaluated by three independent pathologists who were blinded to experimental conditions and groupings. Staining intensity was classified into four categories: negative, weak, moderate, and strong (scores of 0, 1, 2, and 3, respectively), while the extent of staining was scored based on the percentage of positive cells: none, less than 25%, 25%–50%, 50%–75%, and greater than 75% (scores of 0, 1, 2, 3, and 4). The final IHC staining score was calculated by multiplying the intensity score by the range score. For hematoxylin and eosin (H&E) staining, slides underwent a process of dewaxing and rehydration, followed by staining with hematoxylin for 2 min and eosin (Catalog #BL727A, Biosharp) for 1 min, and subsequently dehydrated. Details about the antibodies used for IHC can be found in Table S1.

### 4.9 Immunofluorescence Staining

For cell IF staining, neutrophils (5 × 10^5^) or CT26 cells (4 × 10^4^) were placed on coverslips in 24‐well plates and allowed to adhere for 4 h. They were then fixed using 4% paraformaldehyde for 15 min, followed by two PBS washes. Cells were permeabilized with 0.1% Triton X‐100 for 10 min. After blocking with 10% goat serum for 1 h, the cells were incubated at 4°C overnight with anti‐MPO antibody (1:500, Proteintech, 66,177‐Ig) and anti‐NE antibody (1:500, Abcam, 310335). The next day, cells were rinsed with PBS and incubated with Alexa Fluor 647‐conjugated goat anti‐rabbit secondary antibody (1:500, 4414, CST) or Alexa Fluor 488‐conjugated goat anti‐mouse secondary antibody (1:500, 4408, CST) for 1 h in the dark. After rinsing with PBS, the nuclei underwent staining with DAPI for half an hour at ambient temperature in the dark. After additional PBS rinses, the slides were prepared with an antifade polyvinylpyrrolidone mounting medium (Beyotime, Songjiang, Shanghai, China). The images were acquired using a Leica confocal microscope (Germany).

For tissue IF staining, freshly harvested subcutaneous tumors from mice were fixed in 4% paraformaldehyde for 48 h, followed by sequential immersion in 15% and 30% sucrose for 24 h and embedding in O.C.T. compound. Tumor samples were frozen in liquid nitrogen and sectioned into 8–10 µm slices. The sections were allowed to air dry at ambient temperature for half an hour, then rinsed in PBST for 5 min to eliminate O.C.T. residue. Subsequently, they were fixed with 4% paraformaldehyde for 10 min and underwent antigen retrieval using the Beyotime solution (Cat#P0090) for 20 min. Permeabilization was carried out with 0.5% Triton X‐100 for 10 min, followed by a blocking step using QuickBlock (Cat#P0260, Beyotime) for 20 min. The sections were then incubated with Ly6G (1:100, 238132, Abcam) and citH3 (1:200, 14269S, CST) primary antibodies, followed by secondary antibodies conjugated with Alexa Fluor 488 for anti‐mouse IgG and Alexa Fluor 647 for anti‐rabbit IgG. Finally, tissues were stained with DAPI and examined using a Leica multi‐laser confocal microscope (Germany).

### 4.10 Cell Migration Assay

In vitro migration experiments were conducted utilizing transwell co‐culture chambers with 8 µm pores that were not coated with Matrigel (356,234; Corning; Corning, New York, USA). For the migration experiments, 2 × 10^5^ CRC cells were placed in the upper chamber with serum‐free DMEM, while 1 × 10^6^ neutrophils, either treated with DNase I or untreated, were placed in the lower chamber. Stimuli such as *S. salivarius* or PMA were applied as specified. After 24 h of incubation at 37°C, non‐invading cells were carefully removed, and the cells that migrated to the opposite side of the membrane were fixed with methanol and stained using crystal violet. The stained cells were then counted in five randomly selected fields under an inverted microscope at ×100 magnification.

### 4.11 Cell Proliferation Assay

Cell proliferation was assessed utilizing the Cell Counting Kit‐8 (CCK8) assay. For this experiment, 5,000 cells from the HCT116, SW480, and CT26 lines were initially plated into 96‐well dishes. Six hours post‐seeding, *S. salivarius* was introduced at a multiplicity of infection (MOI) of 100. Following this, the cells were incubated for intervals of 12, 24, 36, 48, and 72 h in the corresponding conditioned medium. Each day, 10 µL of CCK8 solution was added to each well, and absorbance was recorded at 450 nm using a microplate reader (DR‐WIN02 from Wuxi Huawei Delong Instrument Co., Ltd).

### 4.12 Fluorescence In Situ Hybridization (FISH) Assay

FISH assays were performed on paraffin‐embedded tumor sections using the bacterial DNA FISH Assay Kit (c007, GEFANBiology, China). Hybridization was conducted at 37°C for overnight with *S. sanguinis* (5’‐FAM‐ATTTATTGGGCGTAAAGCGAGCGCAGGCGGTTAGAAAAGTCTGAAGT‐3’) or *S. salivarius* (5’‐Cy3‐CACCAATATCTCCATCAATTTCTGCACGAGGTACCAAAGCTGCAA‐3’) probes at 5 µg/mL in hybridization buffer. After hybridization, slides were rinsed with sterile ddH2O, air‐dried in the dark, and mounted with Mounting Medium containing DAPI (ab104139, Abcam, UK). Images were captured using a fluorescent microscope (Leica).

### 4.13 Flow Cytometry Analysis

To evaluate the presence of neutrophils, flow cytometry was employed to analyze the immune cell composition in both blood and tumor tissues. Subcutaneous CT26 tumor tissues were enzymatically digested into single‐cell suspensions using a collagenase‐based dissociation protocol. This solution consisted of Collagenase IV (1 mg/mL; Solarbio, C8160), hyaluronidase (0.1 mg/mL; Solarbio, H8030), and DNase I (0.1 mg/mL; Solarbio, D8071). For each tissue sample, approximately 5 mL of this enzyme solution was prepared. Samples were then incubated at 37°C with continuous shaking at 160 rpm for 1 h to ensure thorough digestion. After digestion, the tumor samples were filtered through a 70 µm cell strainer and centrifuged at 300 g for 5 min. Red blood cells were lysed using a 5‐fold volume of red blood cell lysis buffer (TBD, NH4CL2009), and dead cells were excluded using the Zombie NIR Fixable Viability Kit (BioLegend, 423106). Cells were stained with fluorochrome‐conjugated monoclonal antibodies for cell surface markers (CD45, CD11b, Ly‐6C, or Ly‐6G). Antibodies used are listed in Table S1.

### 4.14 Western Blot

Protein samples were prepared using RIPA buffer (Beyotime, Cat# P0013B) supplemented with protease (Roche, Cat# 11697498001) and phosphatase inhibitors (Roche, Cat# 04906845001). The protein concentration was determined with the BCA Protein Assay Kit (Beyotime, P0012). Proteins underwent separation via 10% SDS‐PAGE and were subsequently transferred onto PVDF membranes (Roche, 3010040001). The membranes were first incubated overnight at 4°C with primary antibodies, then with secondary antibodies for 1 h at ambient temperature. Protein bands were detected using the Omni‐ECL Femto Light Chemiluminescence Kit (MeilunBio, MA0186‐1). GAPDH served as the loading control for total protein, and the antibodies utilized are detailed in Table S1.

### 4.15 Immunoprecipitation (Co‐IP)

Co‐immunoprecipitation experiments were performed using the YEASEN rProtein A/G IP/Co‐IP Kit (Catalog No. 36421ES40). Cell lysates were prepared using the Lysis Buffer for IP Assays provided in the kit, and protein concentrations were determined using the BCA Protein Assay Kit. Protein concentrations were normalized to ensure a total of 1 mg of protein per immunoprecipitation reaction. Equal amounts of lysates were incubated with the target antibody or IgG as a negative control at 4°C with continuous rotation overnight. Pre‐washed Protein A/G MagBeads (IP grade) were then added, and the incubation was continued at 4°C for 12 h to allow the antigen‐antibody complexes to bind to the beads. After incubation, the beads were washed three times with 1 mL of Lysis Buffer using a magnetic stand to remove unbound proteins. Finally, the beads were resuspended in 1× SDS‐PAGE Sample Loading Buffer, heated at 95°C for 5 min, and the supernatant was collected for analysis.

### 4.16 RNA Isolation and Quantitative Real‐Time PCR

Total RNA was extracted using TRIzol reagent (Invitrogen) and reverse transcribed into cDNA with the PrimeScript RT Reagent Kit (Cat#RR047A, TAKARA). Quantitative real‐time PCR was performed using a 7500 Real‐time PCR system (Applied Biosystems) with the FastStart Universal SYBR Green Master Kit (Cat# 04913914001, Roche). Gene expression levels were normalized to Actb expression. Primers used in this assay are listed in Table S2.

### 4.17 5R 16S rRNA Sequencing and Data Analysis

Microbiome profiling was performed using the LC‐Bio 16S RNA sequencing platform. Surgical specimens (tumor [C] and paired adjacent normal tissues [N]) and tongue coating samples (patients with CRC [C_T] pre‐surgery and healthy controls [H_T]) were collected aseptically. All samples were flash‐frozen in liquid nitrogen and stored at −80°C.

DNA extraction was carried out using enzymatic lysis with magnetic bead purification, followed by quality assessment (Nanodrop A260/A280: 1.8‐2.0; Qubit quantification). Five hypervariable regions of the 16S rRNA gene were amplified via multiplex PCR. Libraries were prepared from purified PCR products using AMPure XP beads and sequenced on the Illumina NovaSeq 6000 (250 bp paired‐end).

Raw reads were processed to generate ASVs after quality filtering and denoising. Taxonomic classification was performed using the SILVA database with contaminant removal (negative control cutoff: 0.1%). Alpha diversity (Shannon, Chao1) and beta diversity (PCA) were analyzed using QIIME1. Corresponding data are provided in Figure S1.

### 4.18 Integration of Microbial and Clinical Data

Microbiome‐clinical correlations were analyzed using the standardized workflow on the OECloud platform (Oebiotech Co., Hangzhou, China; https://cloud.oebiotech.com). Key analyses included:

1. Spearman's rank correlation between differentially abundant taxa (at the genus/species level) in tumor/adjacent tissue and the corresponding tongue‐coating microbiota;
2. Association analysis of differential microbial features with clinical phenotypes at the genus/species levels. All statistical computations were performed using the platform's built‐in modules, with significance set a*t p < 0.05*.

### 4.19 Mass Spectrometry Analysis

Protein immunoprecipitation (Co‐IP) was performed using the YEASEN rProtein A/G IP/Co‐IP Kit (Catalog No.36421ES40). Magnetic beads were resuspended, and 40 µL of Protein A/G beads were transferred to a 1.5 mL tube. After lysate preparation (1 mg total protein per reaction), beads were incubated with 5 µg/mL Irgm1 antibody or IgG (negative control) at 4°C overnight with rotation. Beads were washed 3× with 1 mL Lysis Buffer using a magnetic rack, then incubated with protein samples at 4°C overnight to form antibody–protein complexes. After washing 4× with 400 µL Lysis Buffer, bead‐bound proteins were eluted with 1× SDS‐PAGE Sample Loading Buffer (95°C, 5 min) and analyzed by LC‐MS/MS at LUMINGBIO (Shanghai, China). Substrate proteins interacting with Irgm1 were identified based on protein scores and quality metrics.

### 4.20 Transcriptome Sequencing

Neutrophils were collected 4 h post *S.salivarius* treatment, and RNA was extracted using TRIzol reagent. Qualified RNA samples (assessed for quality) were used for library construction and sequencing, performed by OE Biotechnology (Shanghai, China). Differentially expressed genes (DEGs) were defined as *P < 0.05* and *fold change > 1.5*. Hierarchical clustering analysis of DEGs was conducted to validate gene expression patterns across groups and samples. RNA‐seq datasets were further analyzed for differential expression, GO, KEGG, and GSEA.

### 4.21 Single‐Cell RNA‐Seq (DD‐3’)

Single‐cell RNA‐seq libraries were generated using the SeekOne DD Single Cell 3’ Library Preparation Kit (SeekGene, K00202). Cells were mixed with reverse transcription reagents, loaded into chip sample wells, and combined with Barcoded Hydrogel Beads (BHB) and oil phase to form water‐in‐oil droplets. Reverse transcription was performed at 42°C for 90 min, followed by inactivation at 85°C for 5 min. Post‐droplet disruption, cDNA was purified, amplified by PCR, fragmented, end‐repaired, and ligated with sequencing adapters. Libraries were purified using Vazyme DNA purification magnetic beads (N411‐01), quantified by Qubit (Thermo Fisher Scientific, Q33226) and Bioptic Qsep400, and sequenced on the Illumina NovaSeq X Plus platform with paired‐end 150 bp (PE150) reads.

### 4.22 Tissue Dissociation for Single‐Cell Sequencing

Tissues were washed in pre‐cooled PBS (Hyclone, SH30256.01) and dissociated using the Tissue Dissociation Kit A Pro (SeekGene, K01801‐30). Red blood cells were lysed with Solarbio Lysing Solution (R1010). Cell quality was assessed via AO/PI staining and the SeekGene M002C fluorescence cell analyzer, with decisions to remove dead cells (Miltenyi, 130‐090‐101) or debris (Miltenyi, 130‐109‐398) based on viability and nuclear content.CD45^+^ cells were magnetically labeled and sorted using mouse CD45 beads (Miltenyi, 130‐052‐301), then washed twice in RPMI 1640 (Gibco, 11875119) and resuspended in RPMI 1640 containing 2% FBS (Gibco, 10100147C) at 1 × 10^6^ cells/mL.

### 4.23 Statistical Analysis

All quantitative data are presented as mean ± standard deviation (SD). Statistical analyses were performed using GraphPad Prism software (version 10.4.1). Data distributions were assessed for normality and lognormality before statistical testing.

For comparisons between two groups, an unpaired two‐tailed Student's t‐test was used. For comparisons among three or more groups, ordinary one‐way analysis of variance (ANOVA) or two‐way ANOVA was applied as appropriate. Two‐way repeated‐measures ANOVA was used when data involved both time and treatment factors. For datasets with unequal variances, Welch ANOVA was employed. Appropriate post hoc multiple‐comparison tests were applied where necessary.

^*^p < 0.05 was considered statistically significant.

## Author Contributions

Fengyi Liu and Hengxuan Cai designed and wrote the article. Wei Xu organized all the data. Yuan Wang and Xuehan Li analyzed and visualized the results. Zeng Wang performed mechanistic experiments and molecular docking analyses. Xueli Liu and Lai Wei conducted the experiments. Qianwen Yue and Zhihua Zhang analyzed the data. Xuanwen Bao, Fan Xing, Xianjun Li, and Yi Xu provided technical support and critical revisions.

## Funding

This research was supported by the Flagship Department Development Program for Integrated Traditional Chinese and Western Medicine (Guo Zhong Yi Yao Zong Jie Han [2024] No. 221) and the Natural Science Foundation of Heilongjiang Province (Grant No. BS2025H005).

[Correction added 1 March 2026 after online publication: Funding Information is updated in this version.]

## Conflicts of Interest

The authors declare no conflicts of interest.

## Supporting information




**Supporting File 1**: advs74520‐sup‐0001‐SuppMat.docx.

**Supporting File 2**: advs74520‐sup‐0002‐FigureS1‐S6.zip.

**Supporting File 3**: advs74520‐sup‐0003‐TableS1‐S4.docx.

**Supporting File 4**: advs74520‐sup‐0004‐DataFile.pdf.

## Data Availability

The data that support the findings of this study are available from the corresponding author upon reasonable request.
