# Supplementary material for: Oral Streptococcus salivarius Couples Neutrophil IRGM1 Signaling to NET Formation and Colorectal Cancer Metastasis
Source: Adv Sci (Weinh). 2026 Feb 27;13(25):e16546. doi: 10.1002/advs.202516546 (PMC13137785; doi:10.1002/advs.202516546)
Supplement: Supplementary file 1 — Supporting File 1: advs74520‐sup‐0001‐SuppMat.docx. [file ADVS-13-e16546-s004.docx]

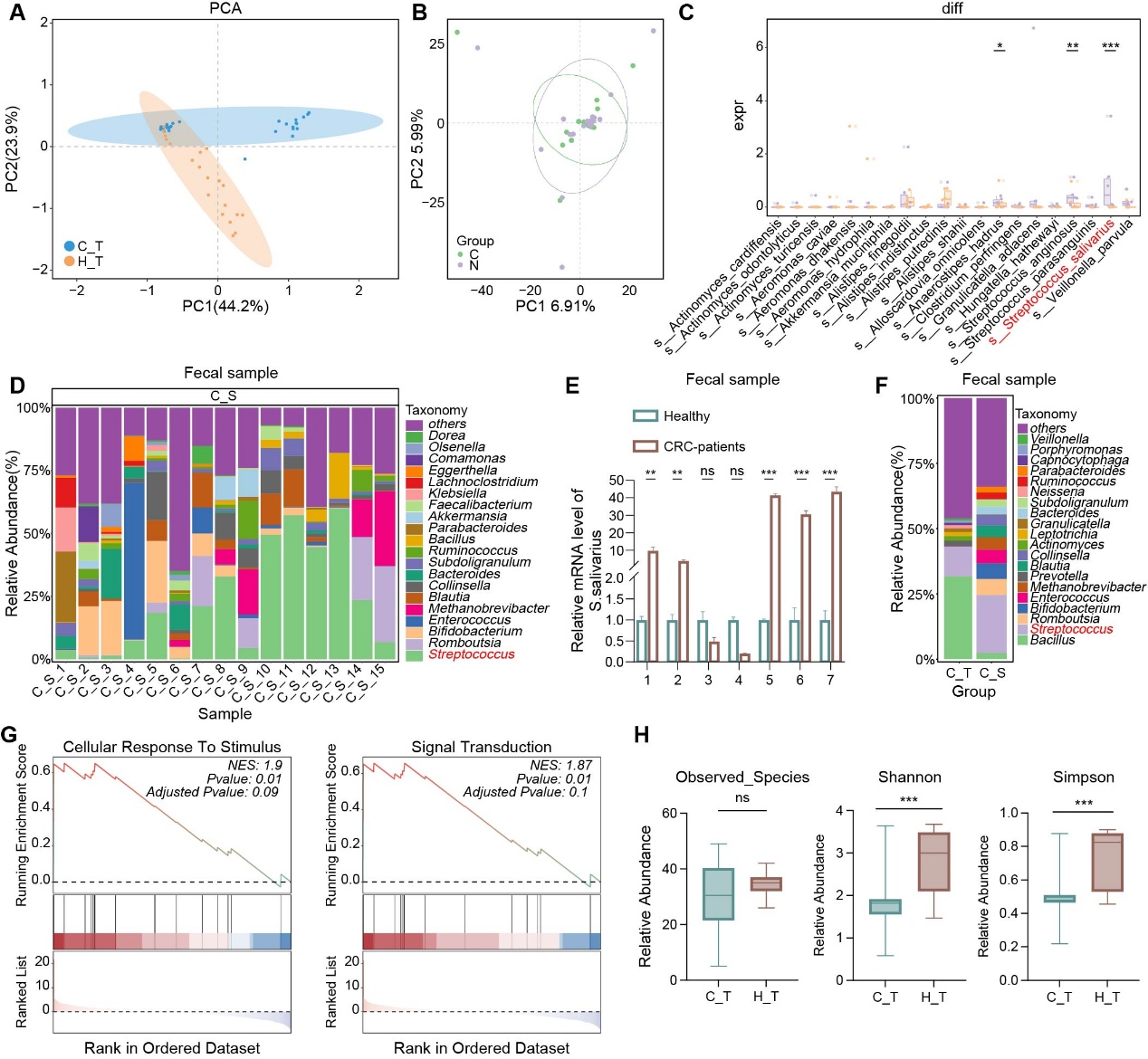


**Figure. S1. Microbial community profiling across oral, fecal, and tumor-associated niches in CRC and healthy individuals.**

(A–B) Beta-diversity analysis based on Bray-Curtis distance using principal component analysis (PCA) and principal coordinate analysis (PCoA), comparing microbial community composition among colorectal cancer tumor tissues. (C) Differential species abundance analysis between tumor (C) and adjacent normal (N) tissues, highlighting enrichment of *S. salivarius* in tumor samples (red). (D) Relative abundance of major taxonomic units in fecal samples from CRC patients (C_S, *n* = 15). (E) qRT–PCR analysis showing increased *S. salivarius* levels in fecal samples from CRC patients compared with healthy controls. (F) Comparison of the relative abundance of major taxonomic units between tongue-coating (C_T) and fecal (C_S) samples from CRC patients. (G) Gene set enrichment analysis (GSEA) showing significant enrichment of Gene Ontology biological process terms related to “response to stimulus” and “signal transduction” in the ranked gene set. (H) Alpha-diversity analysis assessing observed species, Simpson index, and Shannon diversity index. Significant differences in microbial diversity were observed between tumor-associated samples (C_T, *n* = 32) and healthy controls (H_T, *n* = 15). Unclassified taxa were excluded from the analysis. *Data are presented as mean ± SD.* Statistical significance is indicated as**p < 0.05, **p < 0.01, ***p < 0.001.*


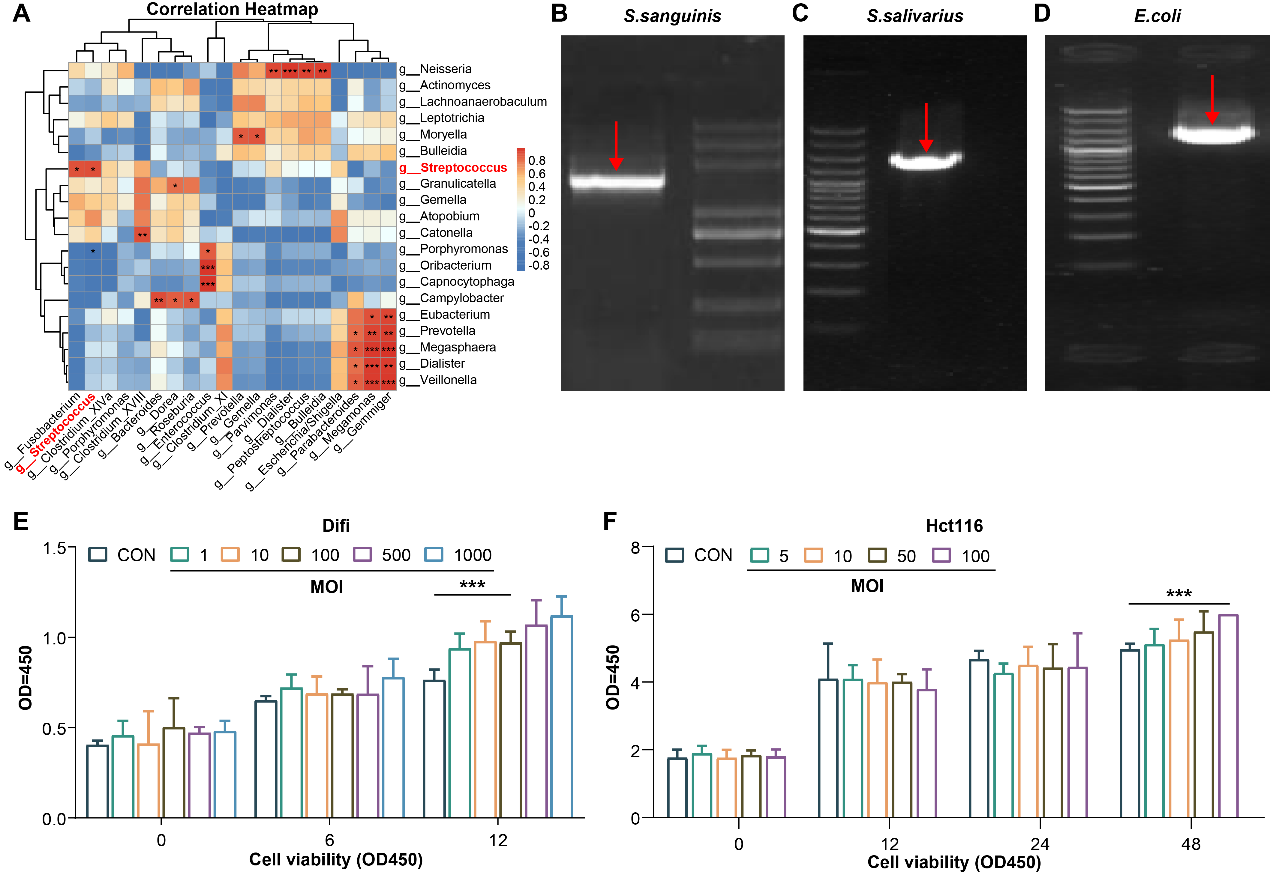


**Figure. S2 Characterization of *S. salivarius* isolates and experimental conditions.**
(A) Heatmaps display correlations between tongue-coating microbiota and tumor tissue microbiota in CRC patients at the genus level. (B–D) PCR-based species identification of *S. sanguinis* (B), *S. salivarius* (C), and *E. coli* (D). Red arrows indicate specific amplification bands confirming strain identity. (E–F) Cell viability assays of Difi (E) and HCT116 (F) cells co-cultured with *S. salivarius* at different multiplicities of infection (MOIs). An MOI of 100 was selected for subsequent experiments based on preserved cell viability. **p < 0.05*, *Data are presented as mean ± SD.*
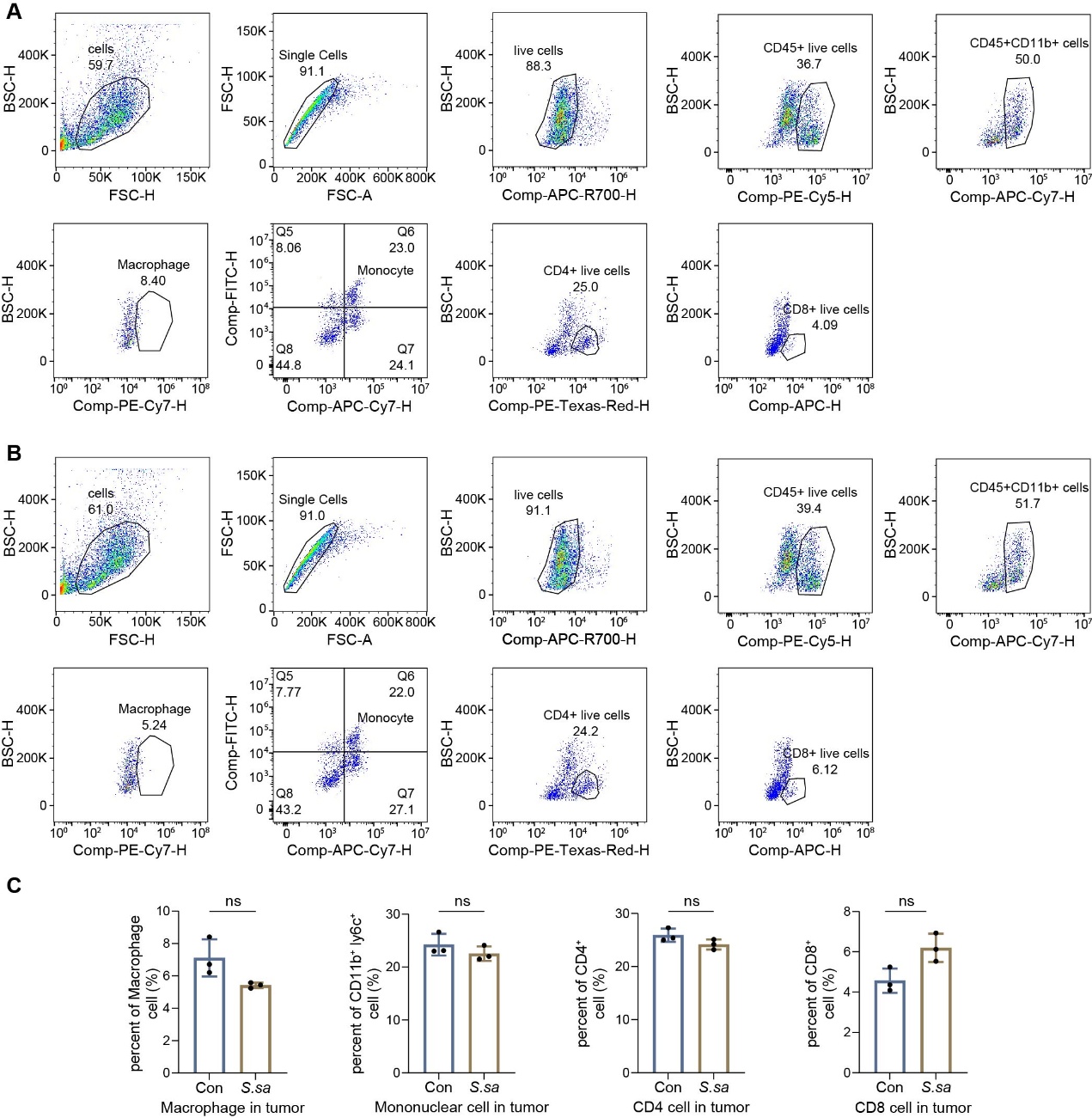
 **Figure. S3 Flow cytometric analysis of immune cell populations in subcutaneous tumors.**

(A) Representative flow cytometry gating strategies for immune cell populations isolated from subcutaneous tumor tissues in control (Con) mice (A) and *Streptococcus salivarius*–treated mice (B). Sequential gating was used to identify total cells, singlets, live cells, CD45⁺ leukocytes, CD45⁺CD11b⁺ myeloid cells, macrophages, monocytes, CD4⁺ T cells, and CD8⁺ T cells. (B) Representative flow cytometry gating strategy for immune cell populations isolated from subcutaneous tumor tissues in *S. salivarius*-treated mice. (C) Quantification of macrophages, monocytes, CD4⁺ T cells, and CD8⁺ T cells among total CD45⁺ cells in subcutaneous tumors from Con and *S. salivarius*–treated mice. Data are presented as mean ± SD. Statistical significance was determined using a two-tailed Mann–Whitney U test. ns, not significant.


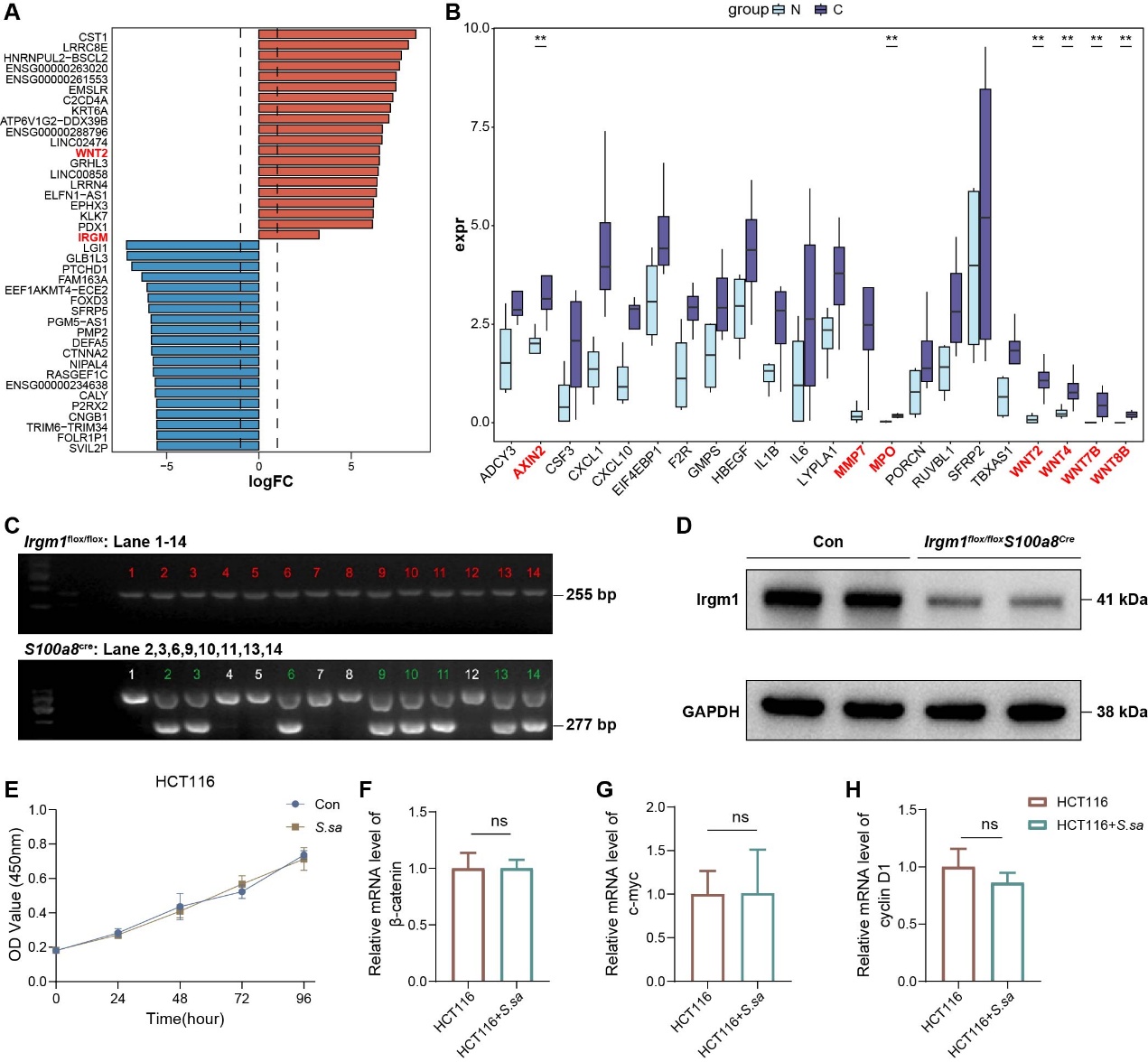


**Figure. S4 Transcriptomic profiling and validation of Irgm1 conditional knockout, and lack of direct Wnt activation in tumor epithelial cells by *S. salivarius*.**

(A) Differentially expressed genes between tumor (C) and adjacent normal (N) tissues from 21 CRC patients. The bar plot shows log fold change (logFC) of significantly upregulated (red, right) and downregulated (blue, left) genes in tumors. Key immune and oncogenic regulators, including *Irgm1* and WNT2, are highlighted in red. (B) Box plots comparing the expression of selected Wnt–signaling–related and neutrophil activation–related genes in tumor versus normal tissues. Genes such as *AXIN2, MMP7, MPO, WNT2, WNT4,* and *WNT7B* were significantly upregulated in tumors (*P < 0.01*, Wilcoxon signed-rank test). (C) PCR-based genotyping. The upper panel shows amplification of the *Irgm1^flox/flox^* allele (255 bp), which was positive in all samples. The lower panel shows the presence of the *S100a8-Cre* allele (277 bp) in samples 2, 3, 6, 9, 10, 11, 13, and 14, confirming successful generation of *Irgm1^flox/flox^S100a8^cre^* mice (*Irgm1-cKO*). (D) Western blot analysis revealed significantly reduced Irgm1 protein expression in *Irgm1-cKO* mice compared to control mice. GAPDH was used as a loading control. The protein samples were extracted from bone marrow neutrophils of *Irgm1-cKO* and C57BL/6 mice. (E) CCK-8 assay showing comparable proliferation of HCT116 cells cultured in the absence or presence of *S. salivarius* over the indicated time course. (F–H) Quantitative analyses showing no significant changes in the expression levels of canonical Wnt/β-catenin pathway effectors, including β-catenin (F), c-Myc (G), and Cyclin D1 (H), in HCT116 cells following *S. salivarius* treatment. Data are presented as mean ± SD from three independent experiments; ns, not significant.


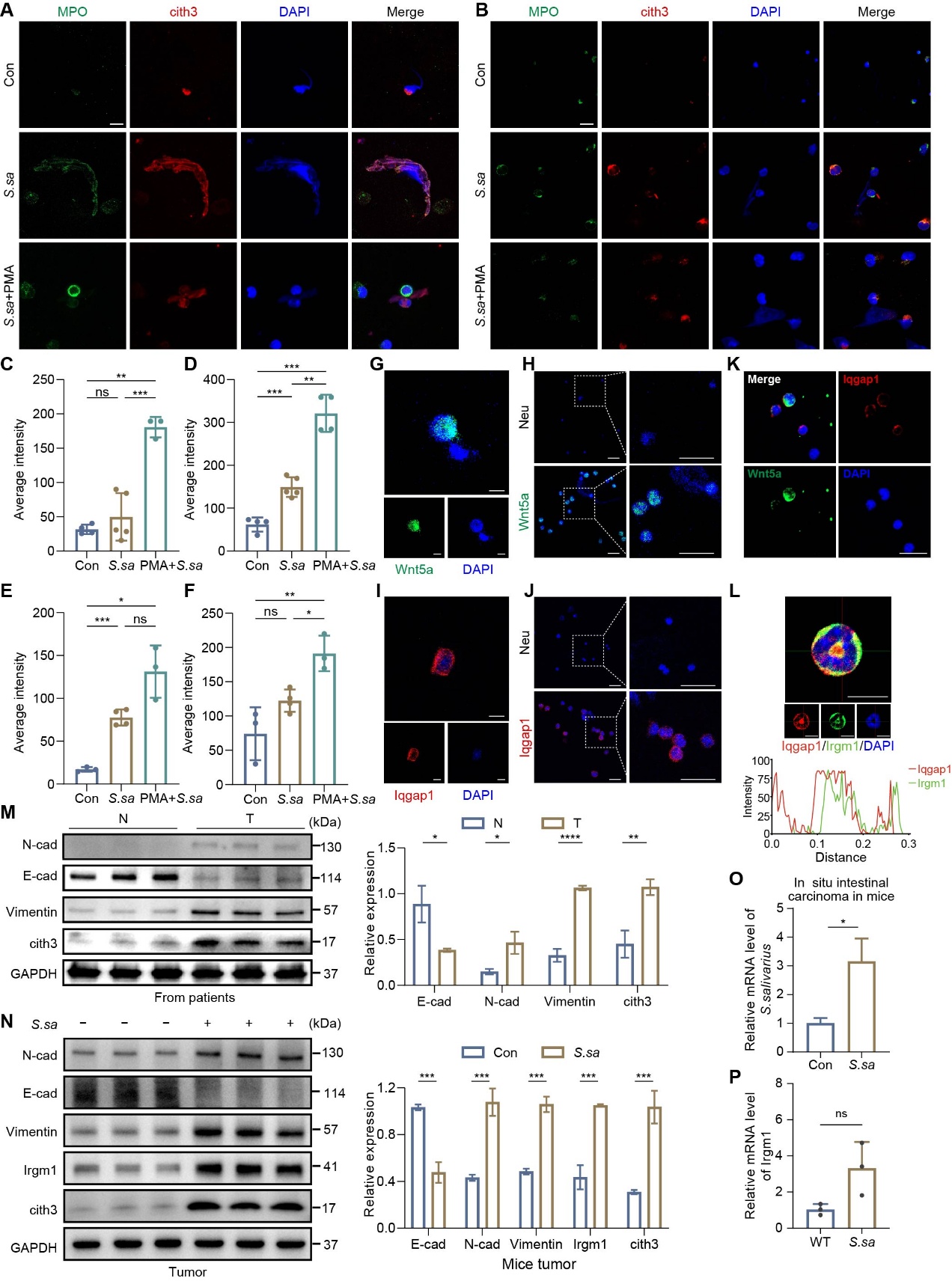


**Figure. S5** ***S. salivarius* promotes NET formation and activates the IRGM1-IQGAP1-Wnt5 axis in neutrophils.**

(A) IF staining showing NET formation in neutrophils from control (Con), *S. salivarius*–treated, and *S. salivarius* + PMA-treated groups. Myeloperoxidase (MPO, green), citrullinated histone H3 (citH3, red), and nuclei (DAPI, blue) were stained. Merged images are shown. Scale bar, 20 μm. (B) IF staining of neutrophil elastase NE (red) and MPO (green) demonstrates their co-localization within NET structures. Nuclei were counterstained with DAPI (blue). (C–F) Quantitative analysis of fluorescence intensity for NETs-associated markers under different treatment conditions:(C) Mean fluorescence intensity of MPO, (D) Co-localization intensity of MPO and citH3;(E) Mean fluorescence intensity of NE, (F) Co-localization intensity of MPO and NE. (G-J) IF analysis showing co-localization of Wnt5a and Iqgap1 with neutrophils, indicating spatial association of Wnt5a and Iqgap1 with neutrophils. (L) IF analysis confirming nuclear co-localization of IRGM1 and IQGAP1. (M) Western blot analysis of E-cadherin, N-cadherin, vimentin, and citH3 expression in CRC patient tumor tissues and matched adjacent normal tissues. (N) Western blot analysis of E-cadherin, N-cadherin, vimentin, Irgm1, and citH3 expression in subcutaneous tumors from mice with or without *S. salivarius* treatment. GAPDH was used as a loading control. (O-P) Quantitative PCR analysis showing absolute quantification of *S. salivarius* and *Irgm1* in orthotopic colorectal tumors from mice. For quantitative analyses, data are presented as mean ± SD from three independent experiments. Statistical significance was determined using Welch ANOVA for immunofluorescence and qPCR analyses, and ordinary one-way ANOVA for western blot quantification. Data normality and lognormality were assessed before statistical testing. **p < 0.05, **p < 0.01, ***p < 0.001.*


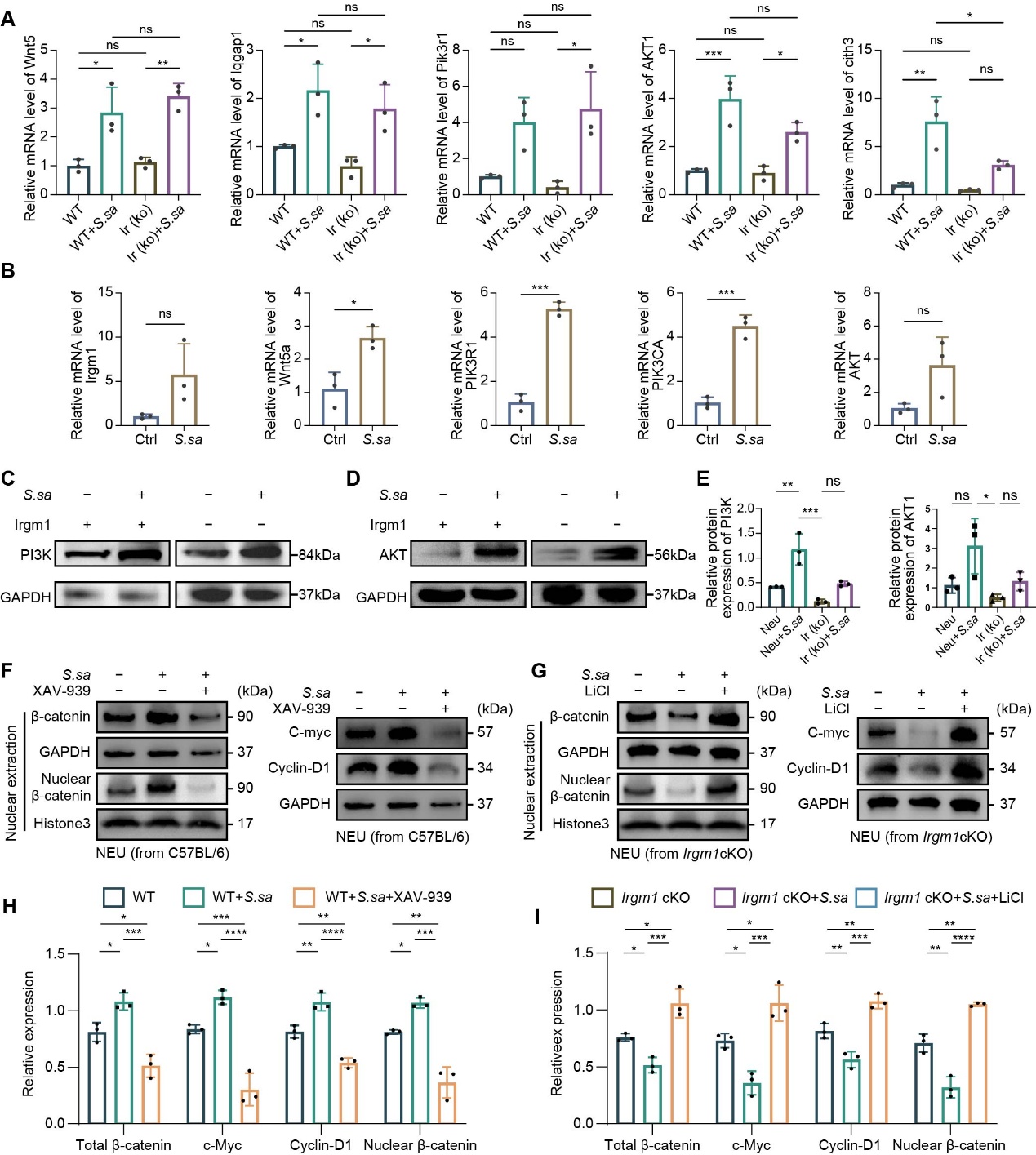


**Figure. S6 IRGM1 is required for *S. salivarius*-associated activation of Wnt5a–PI3K/AKT signaling and NET-related responses in neutrophils**

(A) qRT–PCR analysis of *Wnt5a*, *IQGAP1*, *Pik3r1*, and *CitH3* mRNA expression in lung tissues from wild-type and Irgm1-deficient mice (B) qRT–PCR analysis of *IRGM1*, *Wnt5A*, *PIK3R1*, *PIK3CA*, and *AKT* expression in mouse tumor tissues following *S. salivarius* treatment. (C-D) Representative western blot analyses showing PI3K (C) and AKT (D) protein expression in neutrophils under the indicated conditions. GAPDH was used as a loading control. (E) Densitometric quantification of PI3K and AKT protein levels normalized to GAPDH and expressed relative to the control group. (F) Western blot analysis of β-catenin, c-Myc, Cyclin D1, and nuclear β-catenin levels in wild-type neutrophils treated with *S. salivarius* in the presence or absence of the Wnt pathway inhibitor XAV-939. (G) Western blot analysis of β-catenin signaling components in *Irgm1* conditional knockout neutrophils treated with *S. salivarius* and/or the Wnt pathway activator LiCl. (H) Quantification of total β-catenin, c-Myc, Cyclin D1, and nuclear β-catenin protein levels corresponding to (F). (I) Quantification of β-catenin signaling components corresponding to (G). All quantitative data are presented as mean ± SD from three independent experiments. Statistical significance was determined using an unpaired two-tailed Student’s t-test for two-group comparisons and ordinary one-way ANOVA for comparisons among more than two groups. Data normality was assessed before statistical analysis. **p < 0.05, **p < 0.01, ***p < 0.001.*
