# Supplementary figures and images for: Oral Streptococcus salivarius Couples Neutrophil IRGM1 Signaling to NET Formation and Colorectal Cancer Metastasis
Source: Adv Sci (Weinh). 2026 Feb 27;13(25):e16546. doi: 10.1002/advs.202516546 (PMC13137785; doi:10.1002/advs.202516546)

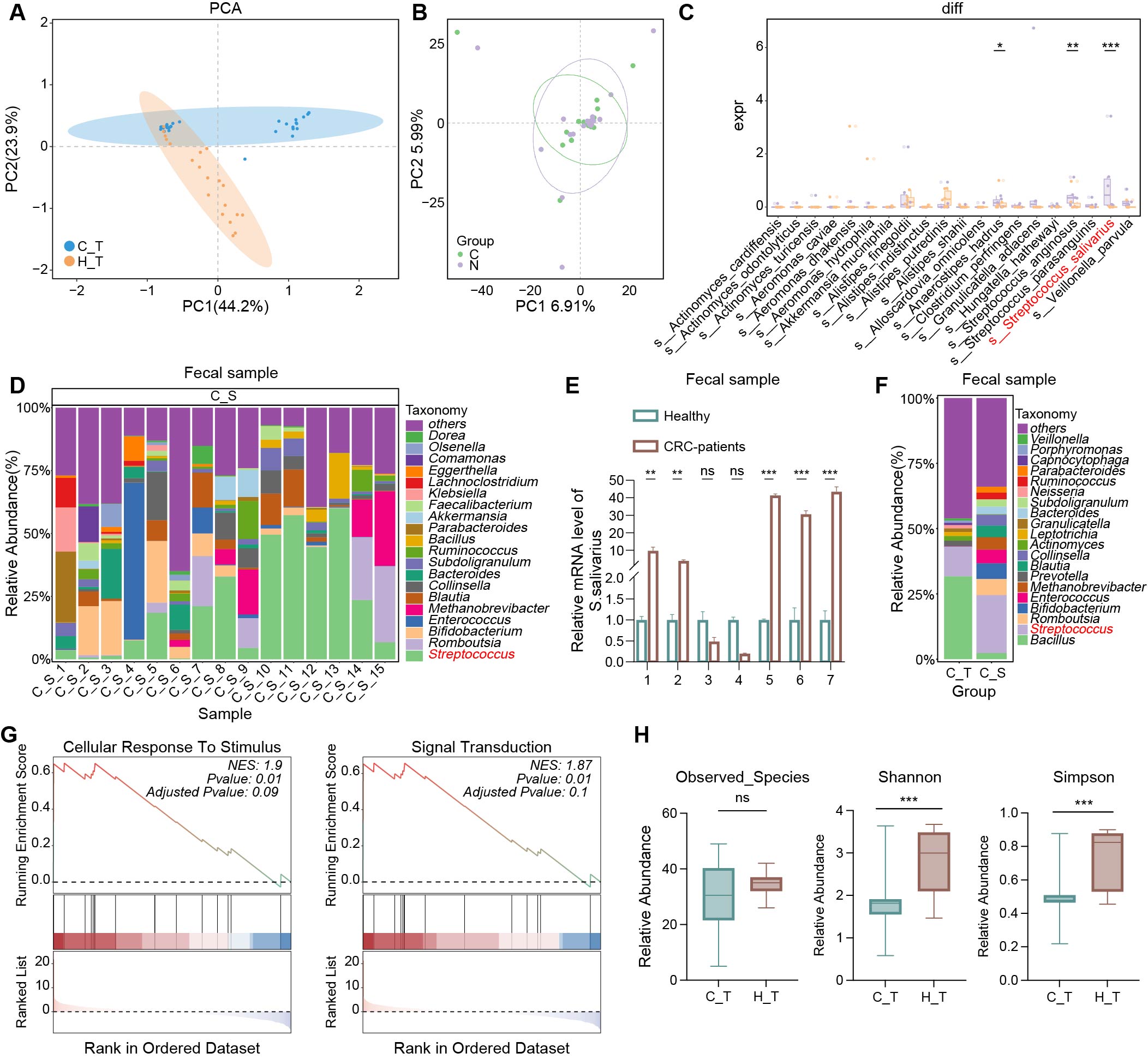

Supplement: Supplementary file 2 — Supporting File 2: advs74520‐sup‐0002‐FigureS1‐S6.zip. [file ADVS-13-e16546-s002.zip › figs1.jpg]

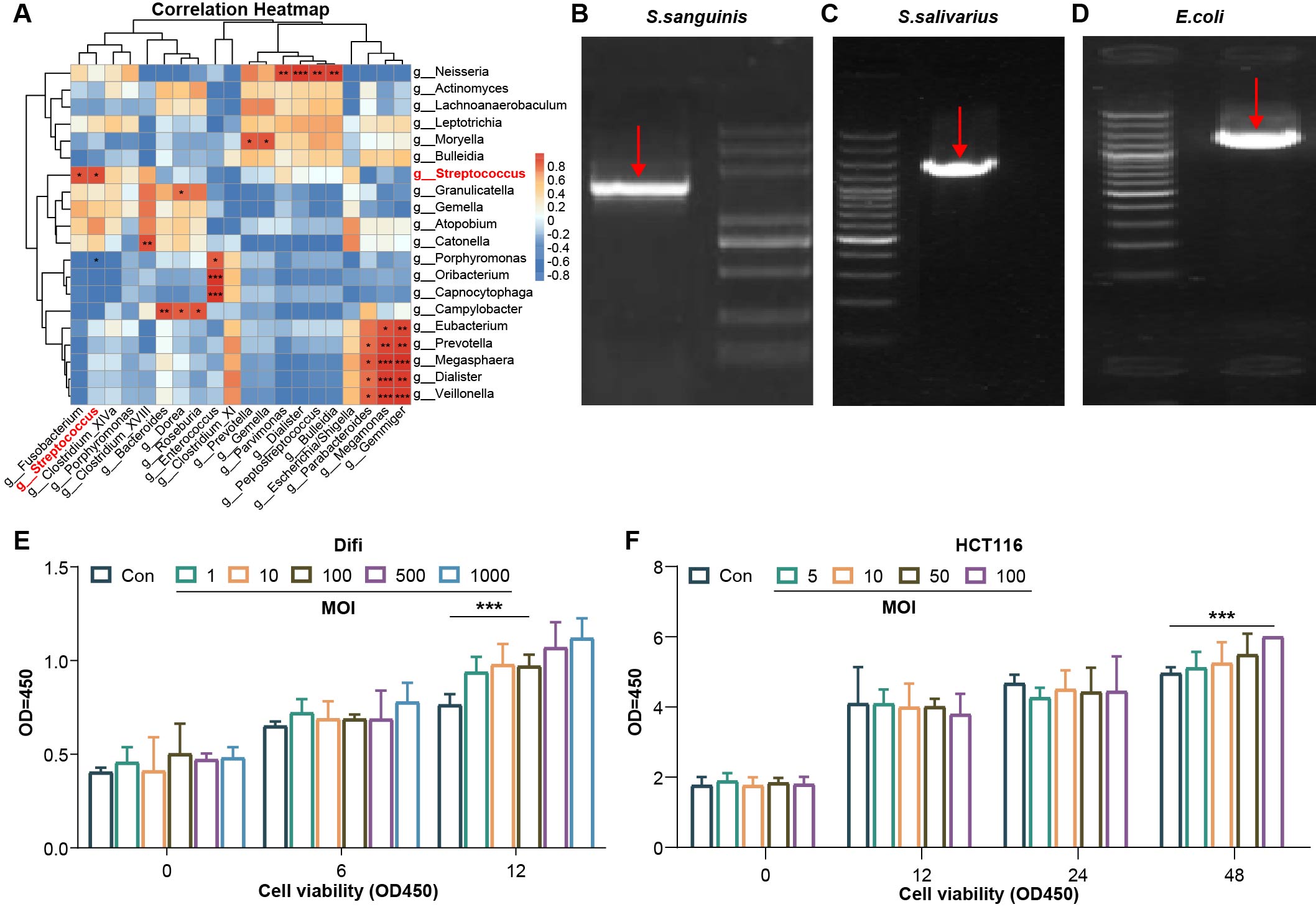

Supplement: Supplementary file 2 — Supporting File 2: advs74520‐sup‐0002‐FigureS1‐S6.zip. [file ADVS-13-e16546-s002.zip › figs2.jpg]

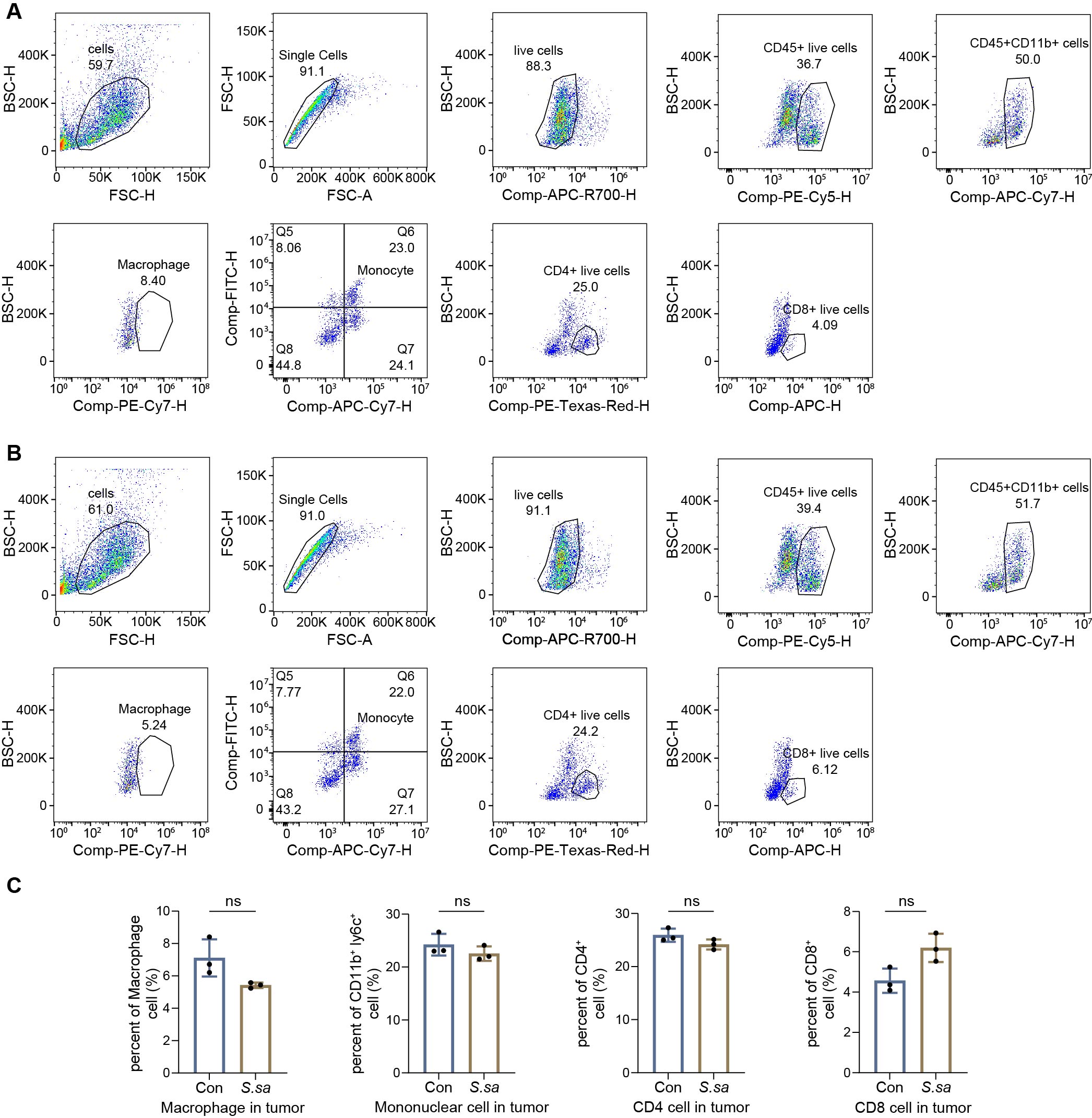

Supplement: Supplementary file 2 — Supporting File 2: advs74520‐sup‐0002‐FigureS1‐S6.zip. [file ADVS-13-e16546-s002.zip › figs3.jpg]

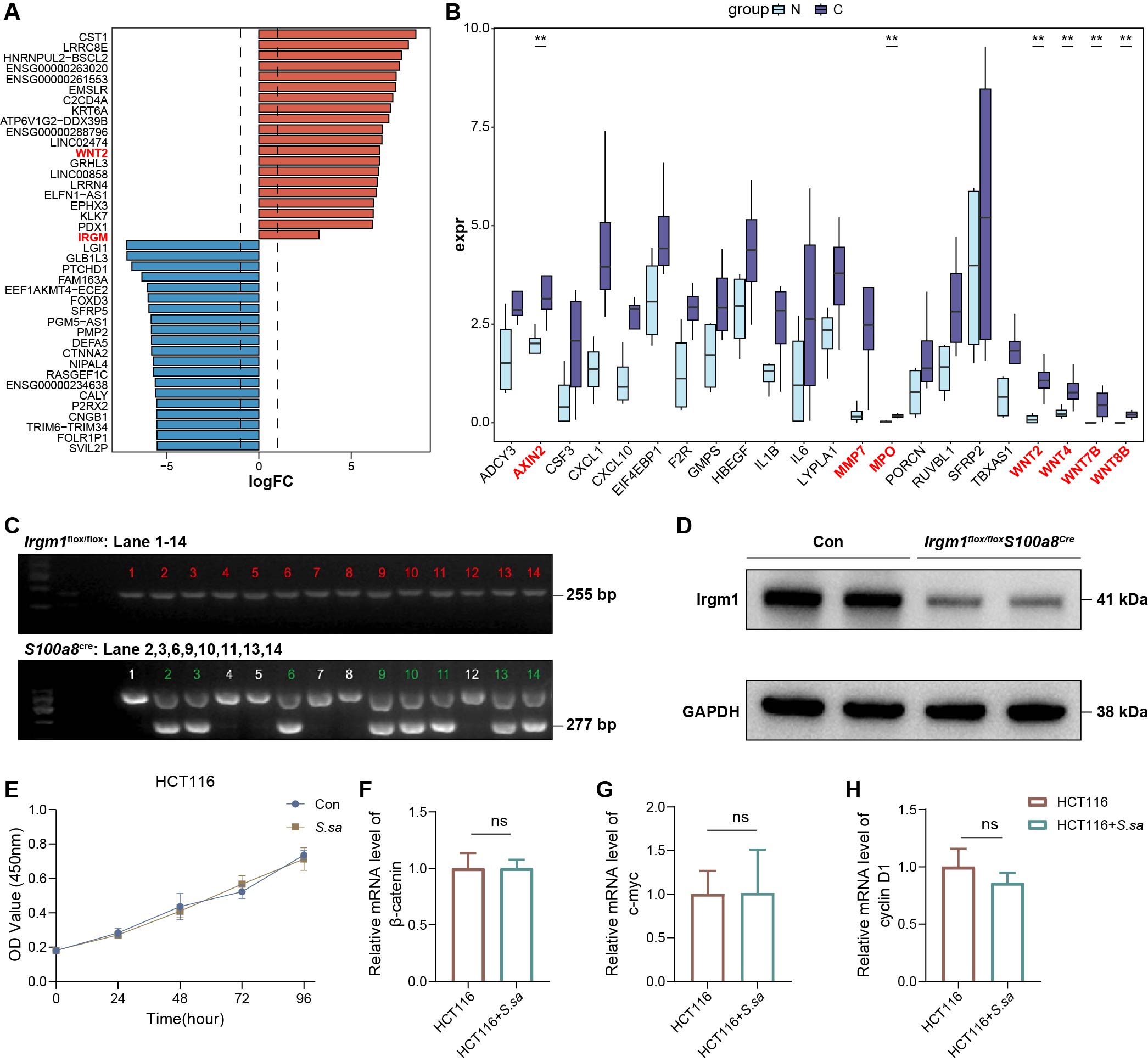

Supplement: Supplementary file 2 — Supporting File 2: advs74520‐sup‐0002‐FigureS1‐S6.zip. [file ADVS-13-e16546-s002.zip › figs4.jpg]

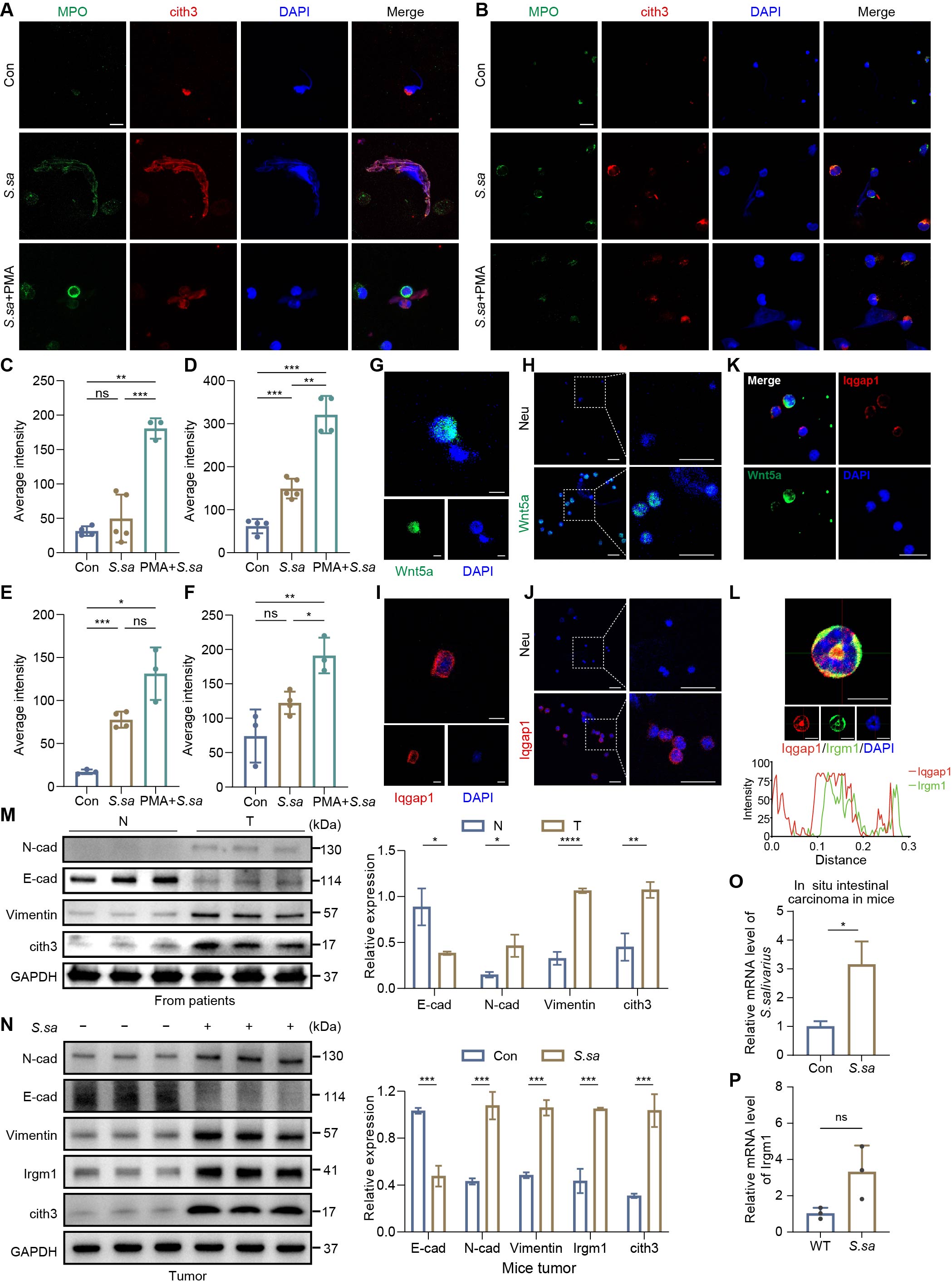

Supplement: Supplementary file 2 — Supporting File 2: advs74520‐sup‐0002‐FigureS1‐S6.zip. [file ADVS-13-e16546-s002.zip › figs5.jpg]

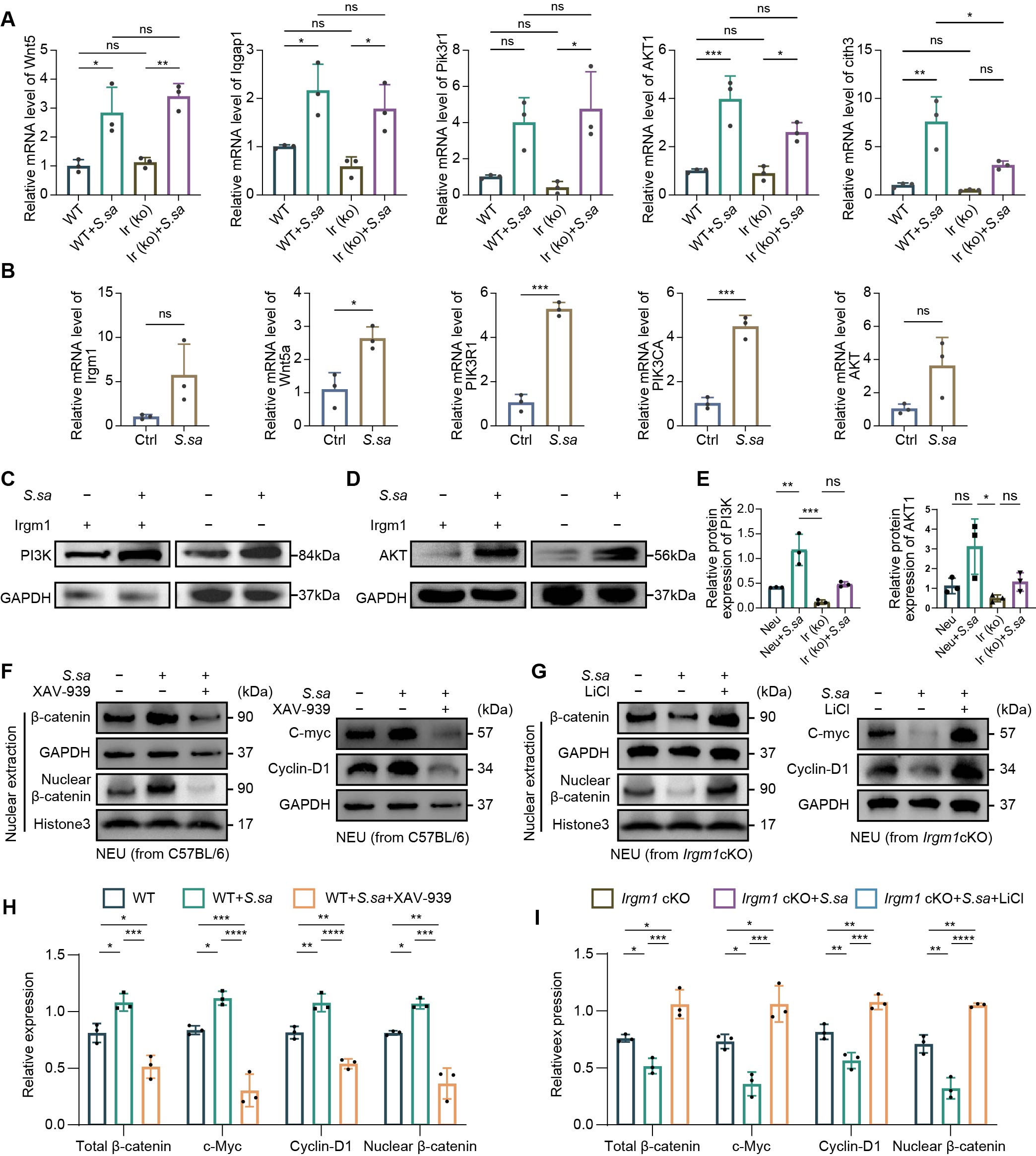

Supplement: Supplementary file 2 — Supporting File 2: advs74520‐sup‐0002‐FigureS1‐S6.zip. [file ADVS-13-e16546-s002.zip › figs6.jpg]

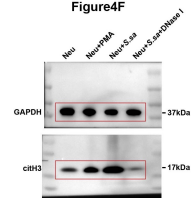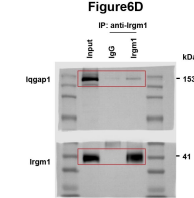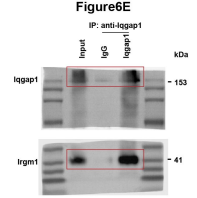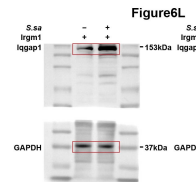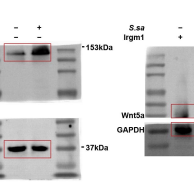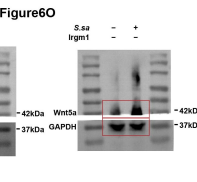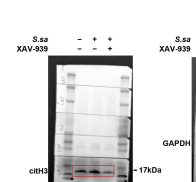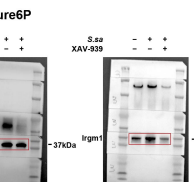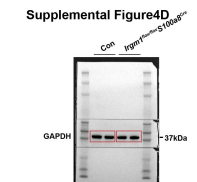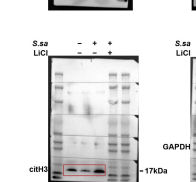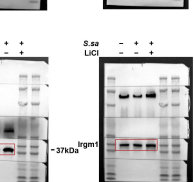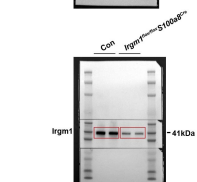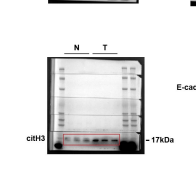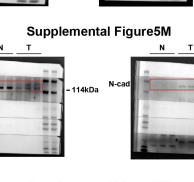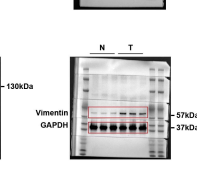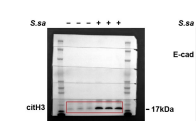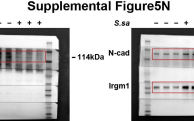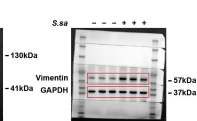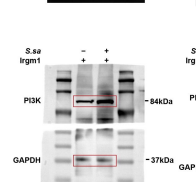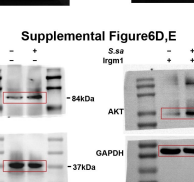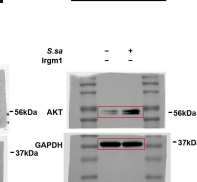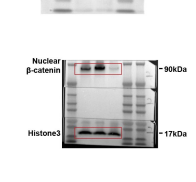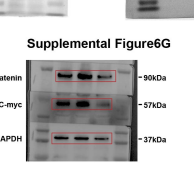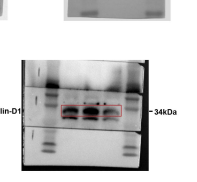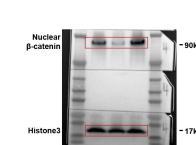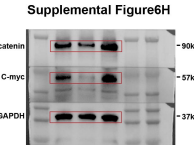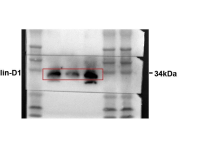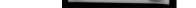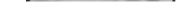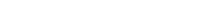

Supplement: Supplementary file 4 — Supporting File 4: advs74520‐sup‐0004‐DataFile.pdf. [file ADVS-13-e16546-s001.pdf]
