# Supplementary material for: Oral Streptococcus salivarius Couples Neutrophil IRGM1 Signaling to NET Formation and Colorectal Cancer Metastasis
Source: Adv Sci (Weinh). 2026 Feb 27;13(25):e16546. doi: 10.1002/advs.202516546 (PMC13137785; doi:10.1002/advs.202516546)
Supplement: Supplementary file 3 — Supporting File 3: advs74520‐sup‐0003‐TableS1‐S4.docx. [file ADVS-13-e16546-s003.docx]

**Supplementary tables**

**Supplementary Table 1**

**Sample clinical and pathologic information**

| **ID** | **Gender** | **Age (years)** | **Pathological type (%)** | **Smoking** | **Diabetes (%)** | **Hypertension (%)** | **Tumor size (cm³)** | **Tstage** | **Nstage** | **Mstage** | **Colon/Rectum (%)** | **Lymphovascular invasion (%)** | **Lymph node metastasis (n)** | **Ki67 (%)** | **CEA (ng/ml)** | **Ferritin (ng/ml)** | **AFP (IU/ml)** | **CA199 (U/ml)** | **NSE (ng/ml)** | **SCC (ng/ml)** | **CA211 (ng/ml)** | **C-reactive protein (mg/L)** |
| --- | --- | --- | --- | --- | --- | --- | --- | --- | --- | --- | --- | --- | --- | --- | --- | --- | --- | --- | --- | --- | --- | --- |
| M1 | Female | 58 | Protruding | no | no | no | 73.5 | 3 | 1 | 0 | Rectum | Not identified | 1 | 0.8 | 17.39 | 13.83 | 1.4 | 58.8 | 17.82 | 0.78 | 3.45 | 81.3 |
| M2 | Female | 63 | Ulcerated-protruding | no | yes | no | 1.105 | 3 | 2 | 0 | Colon | Present | 4 | 0.6 | 17.59 | 9.91 | 2.06 | 124.49 | 17.82 | 0.78 | 2.34 | 46.1 |
| NM3 | Female | 56 | Ulcerative | no | no | no | 2.125 | 3 | 0 | 0 | Colon | Not identified | 0 | 0.75 | 0.95 | 79.45 | 1.46 | 21.09 | 17.82 | 0.78 | 2.3 | 50.34 |
| M4 | Male | 61 | Protruding | yes | no | yes | 11.25 | 3 | 2 | 0 | Rectum | Present | 6 | 0.6 | 4.05 | 634.6 | 1.36 | 212.83 | 17.82 | 0.78 | 4.56 | 56.1 |
| M5 | Male | 59 | Ulcerative | no | no | yes | 5.4 | 3 | 1 | 0 | Colon | Not identified | 2 | 0.85 | 3.45 | 161.70 | 1.04 | 139 | 17.82 | 0.78 | 2.45 | 34.4 |
| M6 | Male | 20 | Ulcerative | no | no | no | 22.5 | 3 | 1 | 0 | Rectum | Not identified | 3 | 0.75 | 21.98 | 51.89 | 1.5 | 97.39 | 25.32 | 0.78 | 6.37 | 50.34 |
| NM7 | Female | 72 | Protruding | yes | yes | no | 63 | 1 | 0 | 0 | Colon | Not identified | 0 | 0.6 | 2.42 | 23.97 | 1.54 | 16.08 | 17.82 | 0.78 | 1.3 | 50.34 |
| NM8 | Male | 64 | Ulcerative | yes | no | no | 9 | 3 | 0 | 0 | Colon | Not identified | 0 | 0.7 | 10.58 | 452.8 | 0.98 | 4.32 | 17.82 | 0.78 | 2.54 | 38.5 |
| M9 | Female | 41 | Ulcerative | no | no | no | 2.5 | 2 | 2 | 0 | Rectum | Present | 7 | 0.85 | 32.05 | 33.08 | 1.77 | 44.45 | 17.82 | 0.78 | 5.67 | 50.34 |
| NM10 | Male | 67 | Protruding | yes | no | no | 18.375 | 3 | 0 | 0 | Rectum | Not identified | 0 | 0.8 | 1.6 | 197.2 | 0.3 | 18.75 | 14.39 | 1 | 3.52 | 50.34 |
| NM11 | Male | 73 | Ulcerative | no | no | no | 9.45 | 3 | 0 | 0 | Colon | Not identified | 0 | 0.7 | 3.2 | 223.3 | 0.98 | 7.5 | 17.82 | 0.78 | 3.12 | 13.4 |
| M12 | Female | 59 | Ulcerative | no | no | no | 4 | 3 | 1 | 0 | Rectum | Not identified | 3 | 0.75 | 19.27 | 132.4 | 1.69 | 195.84 | 12.57 | 0.9 | 2.03 | 50.34 |
| M13 | Female | 47 | Ulcerative | no | no | no | 13.5 | 4 | 2 | 0 | Colon | Present | 14 | 0.85 | 19.22 | 94.32 | 1.48 | 39.74 | 13 | 0.6 | 2.78 | 50.34 |
| NM14 | Male | 64 | Ulcerative | no | no | no | 4.55 | 2 | 0 | 0 | Rectum | Not identified | 0 | 0.4 | 1.26 | 185.9 | 1.62 | 9.31 | 24.37 | 2.8 | 5.95 | 50.34 |
| NM15 | Male | 73 | Ulcerative | no | yes | no | 16 | 3 | 0 | 0 | Rectum | Not identified | 0 | 0.6 | 2.98 | 28.12 | 1.73 | 30.94 | 17.82 | 0.78 | 2.13 | 77.5 |
| NM16 | Female | 74 | Protruding | no | yes | no | 15.75 | 1 | 0 | 0 | Rectum | Not identified | 0 | 0.7 | 1.9 | 61.85 | 0.12 | 10.22 | 17.82 | 0.78 | 1.097 | 50.34 |
| M17 | Male | 53 | Protruding | no | no | no | 6.75 | 3 | 1 | 0 | Rectum | Not identified | 3 | 0.85 | 51.18 | 86.08 | 1.68 | 122.94 | 17.82 | 0.78 | 3.89 | 13.4 |
| M18 | Female | 54 | Ulcerative | no | no | no | 12 | 4 | 1 | 0 | Colon | Present | 1 | 0.85 | 13.14 | 161.70 | 1.83 | 4.38 | 17.82 | 0.78 | 3.24 | 50.34 |
| M19 | Male | 68 | Ulcerative | no | no | yes | 24 | 4 | 1 | 0 | Colon | Present | 2 | 0.75 | 17.42 | 161.70 | 1.76 | 71.92 | 17.82 | 0.78 | 5.34 | 148 |
| NM20 | Female | 77 | Ulcerative | no | no | no | 18 | 2 | 0 | 0 | Rectum | Not identified | 0 | 0.8 | 4.71 | 246.2 | 2.89 | 22.11 | 13.04 | 1.1 | 2.91 | 55.4 |
| NM21 | Male | 66 | Ulcerated-protruding | no | no | no | 27 | 3 | 0 | 0 | Rectum | Not identified | 0 | 0.8 | 8.69 | 54.97 | 1.84 | 47 | 12.62 | 0.5 | 2.15 | 74 |
| NM22 | Male | 68 | Ulcerated-protruding | no | no | yes | 44 | 4 | 0 | 1 | Colon | Present | 0 | 0.75 | 11.26 | 202.6 | 0.52 | 117.4 | 14.28 | 0.7 | 3.55 | 50.34 |
| M23 | Female | 56 | Ulcerative | no | no | no | 35 | 3 | 2 | 0 | Rectum | Present | 6 | 0.85 | 61.87 | 42.52 | 2.43 | 125.12 | 16.19 | 0.4 | 1.59 | 20.3 |
| NM24 | Female | 68 | Ulcerative | no | no | yes | 15.75 | 3 | 0 | 0 | Rectum | Not identified | 0 | 0.6 | 1.45 | 149.4 | 0.97 | 14.52 | 37.03 | 0.3 | 2.29 | 20.9 |
| NM25 | Male | 47 | Ulcerative | no | no | no | 10 | 2 | 0 | 0 | Rectum | Not identified | 0 | 0.8 | 9.3 | 371 | 1.91 | 5.25 | 14.97 | 1.5 | 2.18 | 25 |
| NM26 | Male | 60 | Protruding | no | no | no | 15.75 | 3 | 0 | 0 | Colon | Not identified | 0 | 0.65 | 1.59 | 42 | 2.71 | 11.68 | 21.67 | 0.7 | 2.53 | 38.1 |
| M27 | Female | 68 | Ulcerative | no | no | no | 100 | 3 | 1 | 0 | Colon | Present | 1 | 0.9 | 23.36 | 408.3 | 1.32 | 88.92 | 21.6 | 0.4 | 11.3 | 142 |
| NM28 | Female | 69 | Ulcerative | no | no | no | 6.3 | 3 | 0 | 0 | Colon | Not identified | 0 | 0.75 | 16.51 | 153.9 | 0.26 | 69.45 | 10.45 | 0.6 | 2.29 | 27.9 |
| NM29 | Female | 40 | Ulcerative | no | no | no | 24 | 3 | 0 | 0 | Colon | Not identified | 0 | 0.7 | 3.77 | 1.33 | 1.12 | 8.61 | 8.24 | 0.4 | 5.04 | 21.9 |
| M30 | Male | 35 | Ulcerative | no | no | no | 21 | 3 | 2 | 1 | Junction | Present | 8 | 0.9 | 27.26 | 146 | 0.64 | 1000 | 22.29 | 0.5 | 4.47 | 87 |
| M31 | Male | 36 | Protruding | no | no | no | 8.75 | 2 | 2 | 0 | Colon | Present | 12 | 0.85 | 9.23 | 281.3 | 6.84 | 20.85 | 15.24 | 0.7 | 11.62 | 68.4 |
| NM32 | Male | 59 | Ulcerative | no | no | no | 7.5 | 3 | 0 | 0 | Junction | Not identified | 0 | 0.6 | 2 | 181.2 | 0.16 | 9.11 | 27.47 | 0.6 | 1.28 | 98.8 |
| M33 | Male | 71 | Protruding | no | no | no | 0.75 | 2 | 1 | 0 | Junction | Not identified | 1 | 0.8 | 2.18 | 136.6 | 3.45 | 50.74 | 14.76 | 0.6 | 3.77 | 35.6 |
| M34 | Female | 58 | Ulcerated-protruding | no | no | no | 40 | 3 | 1 | 0 | Junction | Not identified | 2 | 0.8 | 14.24 | 69.03 | 5.7 | 113.42 | 14.13 | 0.3 | 50.6 | 5.61 |
| M35 | Female | 75 | Protruding | no | yes | yes | 55 | 3 | 1 | 0 | Colon | Not identified | 2 | 0.6 | 64.81 | 9.73 | 1.56 | 45.82 | 19.55 | 0.5 | 3.46 | 65.9 |
| NM36 | Female | 74 | Protruding | no | no | yes | 3.24 | 3 | 0 | 0 | Rectum | Present | 0 | 0.65 | 14.4 | 91.88 | 0.86 | 23.78 | 17.22 | 0.4 | 2.5 | 50.34 |
| NM37 | Female | 57 | Protruding | no | no | yes | 148.5 | 3 | 0 | 0 | Colon | Not identified | 0 | 0.7 | 3.93 | 6.55 | 1.86 | 8.77 | 12.29 | 0.7 | 2.39 | 50.34 |
| NM38 | Female | 52 | Ulcerative | no | no | yes | 24.3 | 3 | 0 | 0 | Rectum | Present | 0 | 0.7 | 17.42 | 161.70 | 0.76 | 51.92 | 17.82 | 0.78 | 1.02 | 50.34 |
| M39 | Male | 70 | Ulcerative | no | no | no | 17.55 | 3 | 1 | 0 | Colon | Present | 3 | 0.75 | 297.7 | 46.27 | 2.86 | 1000 | 31.62 | 0.5 | 3.57 | 44.2 |
| M40 | Female | 53 | Ulcerative | no | yes | no | 15.75 | 3 | 1 | 0 | Rectum | Not identified | 1 | 0.85 | 9.66 | 58.56 | 0.93 | 50.65 | 13.52 | 0.7 | 2.15 | 50.34 |
| NM41 | Male | 62 | Ulcerative | no | yes | yes | 7.5 | 3 | 0 | 0 | Rectum | Not identified | 0 | 0.7 | 17.42 | 161.70 | 0.76 | 41.92 | 17.82 | 0.78 | 1.22 | 97 |
| NM42 | Male | 69 | Protruding | no | no | no | 3.75 | 2 | 0 | 0 | Rectum | Not identified | 0 | 0.5 | 1.83 | 348.7 | 0.8 | 15.3 | 9.02 | 1.1 | 4.2 | 23.6 |
| M43 | Female | 41 | Ulcerative | no | no | no | 18 | 3 | 2 | 0 | Junction | Not identified | 4 | 0.85 | 12.39 | 1.09 | 1.79 | 9.04 | 12.89 | 0.5 | 1.81 | 21 |
| M44 | Male | 67 | Ulcerative | no | no | no | 15.75 | 3 | 1 | 0 | Colon | Not identified | 2 | 0.7 | 17.42 | 161.70 | 1.76 | 71.92 | 17.82 | 0.78 | 5.46 | 5.57 |
| M45 | Male | 67 | Ulcerative | no | no | yes | 9 | 3 | 2 | 0 | Rectum | Not identified | 6 | 0.7 | 22.43 | 327 | 1.86 | 7.82 | 11.62 | 1 | 2.14 | 37.5 |
| M46 | Male | 75 | Ulcerated-protruding | no | yes | yes | 65.2 | 3 | 1 | 0 | Junction | Not identified | 1 | 0 | 4.26 | 784.6 | 1.93 | 89 | 24.28 | 1.6 | 2.32 | 59.4 |
| NM47 | Male | 60 | Protruding | no | no | no | 1.8 | 1 | 0 | 0 | Colon | Not identified | 0 | 0.7 | 1.26 | 160.5 | 0.94 | 12.43 | 28.98 | 0.9 | 2.05 | 27.3 |
| H1 | Female | 46 | Normal colorectal mucosa |  |  |  |  |  |  |  |  |  |  |  |  |  |  |  |  |  |  |  |
| H2 | Female | 60 | Colonic polyp |  |  |  |  |  |  |  |  |  |  |  |  |  |  |  |  |  |  |  |
| H3 | Female | 48 | Normal colorectal mucosa |  |  |  |  |  |  |  |  |  |  |  |  |  |  |  |  |  |  |  |
| H4 | Male | 48 | Normal colorectal mucosa |  |  |  |  |  |  |  |  |  |  |  |  |  |  |  |  |  |  |  |
| H5 | Female | 63 | Normal colorectal mucosa |  |  |  |  |  |  |  |  |  |  |  |  |  |  |  |  |  |  |  |
| H6 | Male | 58 | colonic polyps, colitis |  |  |  |  |  |  |  |  |  |  |  |  |  |  |  |  |  |  |  |
| H7 | Male | 46 | Colonic polyp |  |  |  |  |  |  |  |  |  |  |  |  |  |  |  |  |  |  |  |
| H8 | Male | 53 | Colonic polyp |  |  |  |  |  |  |  |  |  |  |  |  |  |  |  |  |  |  |  |
| H9 | Female | 59 | Melanosis coli |  |  |  |  |  |  |  |  |  |  |  |  |  |  |  |  |  |  |  |
| H10 | Male | 54 | Normal colorectal mucosa |  |  |  |  |  |  |  |  |  |  |  |  |  |  |  |  |  |  |  |
| H11 | Female | 46 | Normal colorectal mucosa |  |  |  |  |  |  |  |  |  |  |  |  |  |  |  |  |  |  |  |
| H12 | Male | 40 | Normal colorectal mucosa |  |  |  |  |  |  |  |  |  |  |  |  |  |  |  |  |  |  |  |
| H13 | Male | 61 | Normal colorectal mucosa |  |  |  |  |  |  |  |  |  |  |  |  |  |  |  |  |  |  |  |
| H14 | Male | 52 | Normal colorectal mucosa |  |  |  |  |  |  |  |  |  |  |  |  |  |  |  |  |  |  |  |

**Supplementary Table 2. The comparison and analysis of clinical indicators between the Non-metastasis and Metastasis groups**

| **Index** | **Non-metastasis (n=23)** | **Metastasis (n=24)** | **P value (*P<0.05)** |
| --- | --- | --- | --- |
| Age (years) | 66.00 (59.00-72.00) | 58.50 (48.50-67.75) | 0.0526 |
| Tumor size (cm³) | 15.75 (6.30-24.00) | 15.75 (7.25-32.25) | 0.6018 |
| Lymph node metastasis (n) | 0.00 (0.00-0.00) | 3.00 (1.25-6.00) | 0.0000* |
| Ki67 (%) | 70.00 (60.00-75.00) | 80.00 (71.25-85.00) | 0.0016* |
| CEA (ng/ml) | 3.20 (1.60-10.58) | 17.51 (10.34-26.29) | 0.0000* |
| Ferritin (ng/ml) | 160.50 (54.97-202.60) | 113.36 (43.46-161.71) | 0.4689 |
| AFP (IU/ml) | 0.98 (0.76-1.73) | 1.760 (1.420-2.03) | 0.0037* |
| CA199 (U/ml) | 15.30 (9.11-30.94) | 80.42 (44.79-124.96) | 0.0001* |
| NSE (ng/ml) | 17.82 (13.04-17.82) | 17.82 (14.29-19.12) | 0.6471 |
| SCC (ng/ml) | 0.78 (0.60-0.90) | 0.78 (0.5-0.78) | 0.3287 |
| CA211 (ng/ml) | 2.30 (2.05-3.12) | 3.52 (2.33-5.43) | 0.0103* |
| C-reactive protein (mg/L) | 50.35 (27.30-50.35) | 50.355 (34.70-64.28) | 0.8035 |
| Procalcitonin (ng/ml) | 0.42 (0.19-0.42) | 0.42 (0.14-0.45) | 0.6959 |
| Gender (%) |  |  | 0.6542 |
| Male | 56.50% | 50.00% |  |
| Female | 43.50% | 50.00% |  |
| Smoking (%) |  |  | 0.2756 |
| Yes | 13.00% | 4.20% |  |
| No | 87.00% | 95.80% |  |
| Diabetes (%) |  |  | 0.9473 |
| Yes | 17.40% | 16.70% |  |
| No | 82.60% | 83.30% |  |
| Hypertension (%) |  |  | 0.9319 |
| Yes | 26.10% | 25.00% |  |
| No | 73.90% | 75.00% |  |
| T stage (%) |  |  | 0.2217 |
| T1 | 13.00% | 0.00% |  |
| T2 | 17.40% | 12.50% |  |
| T3 | 65.20% | 75.00% |  |
| T4 | 4.30% | 12.50% |  |
| N stage (%) |  |  | 0.0000* |
| N0 | 100% | 0 |  |
| N1 | 0 | 62.50% |  |
| N2 | 0 | 37.50% |  |
| M stage (%) |  |  | 0.9755 |
| M0 | 95.70% | 95.80% |  |
| M1 | 4.30% | 4.20% |  |
| Pathological type (%) |  |  | 0.7380 |
| Ulcerated-protruding | 8.70% | 12.50% |  |
| Ulcerative | 56.50% | 62.50% |  |
| Protruding | 34.80% | 25.00% |  |
| Colon/Rectum (%) |  |  | 0.2149 |
| Junction (rectosigmoid) | 4.30% | 20.80% |  |
| Colon | 43.50% | 41.70% |  |
| Rectum | 52.20% | 37.50% |  |
| Lymphovascular invasion (%) |  |  | 0.0140* |
| Present | 13.00% | 45.80% |  |
| Not identified | 87.00% | 54.20% |  |

**Supplementary Table 3. The Reagents used in this study.**

| Antibodies | Source | Identifier |
| --- | --- | --- |
| Rabbit Anti-Vimentin antibody | SANTA | sc-6260 |
| E Cadherin (CDH1) Rabbit Polyclonal Antibody | ORIGENE | TA321651S |
| N-Cadherin (CDH2) Mouse Monoclonal Antibody | ORIGENE | TA503933S |
| Beta Catenin Polyclonal antibody | Proteintech | 51067-2-AP |
| Cyclin D1 Monoclonal antibody | Proteintech | 60186-1-lg |
| Anti-c-Myc antibody | GeneTex | GTX103436 |
| Anti-Irgm1 antibody | GeneTex | GTX85039 |
| Irgm1 (E6P7W) Rabbit mAb | CST | E6P7W |
| Anti-Histone H3 (citrulline R2 + R8 + R17) antibody | Abcam | ab281584 |
| Mouse Anti-GAPDH mAb | ZSGB-BIO | TA-08 |
| Histone H3 Polyclonal antibody | Proteintech | 17168-1-AP |
| Goat Anti-Mouse IgG | ZSGB-BIO | ZB-2305 |
| Goat Anti-rabbit IgG | ZSGB-BIO | ZB-2301 |
| MPO antibody | Proteintech | 66177-1-Ig |
| Irgm1 antibody | GeneTex | GTX85038 |
| Anti-Neutrophil Elastase antibody | Abcam | ab310335 |
| Histone H3 Antibody | Novus Biologicals | NB100-57135 |
| MPO Monoclonal antibody | Proteintech | 66177-1-Ig |
| Histone H3 (1B1B2) Mouse mAb | CST | CST-14269S |
| Anti-Neutrophil Elastase antibody | Abcam | ab310335 |
| Anti-Ly6g antibody | Abcam | ab238132 |
| Anti-mouse IgG AF488 | CST | 4408 |
| Anti-rabbit IgG AF647 | CST | 4414 |
| FITC anti-mouse CD45 Antibody | Biolegend | 157214 |
| APC anti-mouse/human CD11b Antibody | Biolegend | 101212 |
| PE anti-mouse Ly-6G Antibody | Biolegend | 127608 |
| PerCP anti-mouse Ly-6C Antibody | Biolegend | 128027 |
| Zombie Aqua™ Fixable Viability Kit | Biolegend | 423101 |
| Rabbit polyclonal antibody to Wnt5a | Affinity | DF6856 |
| Anti-PI 3 Kinase p85 alpha Antibody | HUABIO | ET1608-70 |
| CITH3 antibody | Wanleibio | WL09840a |
| Rabbit Monoclonal to AKT1/2/3 | HUABIO | ET1609-51 |
| IQGAP1 antibody | Santa Cruz | SC-376021 |
| IRGM1 antibody | R&D Systems | mAb71950 |
| PE/Cyanine7 anti-mouse F4/80 | BioLegend | 123113 |
| PE anti-mouse Ly-6G | MultiSciences | FMPLY6G-01-025 |
| APC-Cy7 anti-human/mouse D11b | MultiSciences | FHO011b-02-025 |
| Purified anti-mouse CD16/32 | MultiSciences | FMU16/32-02-100 |
| PerCP-Cy5.5 anti-mouse | MultiSciences | FMS045-01-025 |
| CD11b polyclonal antibody | Proteintech | 31745-1-AP |
| IRGM1 | Affinity | DF13821 |
| Mouse Guanylated Histone H3(CITH3) | J&L Biological | JL48480 |
| Mouse MPO (Myeloperoxidase) | Elabscience | E-EL-M3125 |

**Supplementary Table 4. The sequence of primers used in this study.**

| Primer | Sequence (5’->3’) |
| --- | --- |
| Mouse NE forward | CCTTGGCAGACTATCCAGCC |
| Mouse NE reverse | GACATGACGAAGTTCCTGGCA |
| Mouse MPO forward | AGGGCCGCTGATTATCTACAT |
| Mouse MPO reverse | CTCACGTCCTGATAGGCACA |
| Mouse PAD4 forward | TCTGCTCCTAAGGGCTACACA |
| Mouse PAD4 reverse | GTCCAGAGGCCATTTGGAGG |
| Mouse MMP9 forward | GCAGAGGCATACTTGTACCG |
| Mouse MMP9 reverse | TGATGTTATGATGGTCCCACTTG |
| Mouse Irgm1 forward | TGGCAATGGCATGTCATCTT |
| Mouse Irgm1 reverse | AGTACTCAGTCCGCGTCTTCGT |
| Mouse β-actin forward | GGCTGTATTCCCCTCCATCG |
| Mouse β-actin reverse | CCAGTTGGTAACAATGCCATGT |
| *S. salivarius* primer 1F | TGCTGGAACTAAACGGTAGC |
| *S. salivarius* primer 1R | CAACGACTTCAGAGCCTCCC |
| *S. salivarius* primer 2F | CCTCACGGCGGTCTTTACTCAA |
| *S. salivarius* primer 2R | CGGTACTTTATCGGCCTTGTGCT |
| Mus musculus Wnt5a -F | TCGCAGAAACATTTTAGACACCT |
| Mus musculus Wnt5a -R | AAAACCACCGTCCTAACATCAG |
| Mus musculus Pik3r1 -F | TATTGGCAGGGGAAGCGAGAC |
| Mus musculus Pik3r1 -R | CTTGACTTCGCGTCTACCA |
| Mus musculus Akt1 NM-F | GATAACGGACTTCGGTGGTGT |
| Mus musculus Akt1 NM-R | CGGCCACACATCATCTCGTA |
| Iqgap1‑F | ACCAGAGTGACCTTGCTGAAGC |
| Iqgap1‑R | TTGCGTCTCCAGGTTGTGGTAG |
| Pik3ca‑F | CACCTGAACAGACAAGTAGAGGC |
| Pik3ca‑R | GCAAAGCATCCATGAAGTCTGGC |
